# Ti_3_C_2_T*_x_* MXene‐Zirconium Diboride Based Ultra‐High Temperature Ceramics

**DOI:** 10.1002/advs.202500487

**Published:** 2025-04-30

**Authors:** Srinivasa Kartik Nemani, Nicola Gilli, Steven Goldy, Ankit Kumar, Yooran Im, Austin J. Vorhees, Brian C. Wyatt, Nithin Chandran BS, Nikhilesh Chawla, Garritt J. Tucker, Laura Silvestroni, Babak Anasori

**Affiliations:** ^1^ School of Mechanical Engineering Purdue University West Lafayette IN 47907 USA; ^2^ CNR‐ISMN Institute for Nanostructured Materials Via Gobetti 101 Bologna 40129 Italy; ^3^ Colorado School of Mines Golden CO 80401 USA; ^4^ School of Materials Engineering Purdue University West Lafayette IN 47907 USA; ^5^ Department of Physics Baylor University Waco TX 76706 USA; ^6^ CNR‐ISSMC Institute of Science, Technology and Sustainability for Ceramics Via Granarolo 64 Faenza 48018 Italy

**Keywords:** core–shell structure, dislocations, MXenes, spark plasma sintering, UHTCs, zirconium diboride

## Abstract

MXenes are a family of two‐dimensional (2D) transition metal carbides, nitrides, and carbonitrides with potential applications in ceramics and composites due to their nanometer‐thick morphology, hydrophilic surfaces, and negative zeta potentials. In this study, we investigated titanium carbide MXene (Ti_3_C_2_T*_x_*) as an additive in ultra‐high‐temperature ceramics (UHTCs), specifically zirconium diboride (ZrB_2_). Homogeneous green bodies of Ti_3_C_2_T*_x_* and ZrB_2_ were synthesized via electrostatic self‐assembly in aqueous media without surfactants and subsequently densified using field‐assisted (spark plasma) sintering. The incorporation of 0.5 wt.% MXene enhanced the relative density of ZrB_2_ from ≈89% (pure ZrB_2_) to ≈96% under identical sintering conditions. MXene addition significantly reduced the oxygen content from ≈5 at.% in pure ZrB₂ to ≈2–3 at.% at 2.5 wt.% MXene loading. The presence of MXene also facilitates the formation of a core–shell microstructure, where (Zr,Ti)B_2_ shells encapsulate ZrB₂ cores, with arrays of dislocations observed at the core–shell interface. Mechanical characterizations show substantial improvements, including a 36% increase in hardness, a ≈12% enhancement in Young's modulus, and a ≈15% increase in flexural strength at 2.5 wt.% MXene loading. These findings demonstrate the potential of MXenes as effective sintering aids and reinforcement agents in UHTCs, offering promising pathways for advancing materials designed for extreme environments.

## Graphical abstract

This study demonstrates the effectiveness of Ti_3_C_2_T*_x_* MXenes as additives in ultra‐high‐temperature ceramics (ZrB₂), enhancing densification, reducing oxygen content, and improving mechanical properties. Through surfactant‐free electrostatic self‐assembly and spark plasma sintering, MXenes enabled the formation of a core–shell microstructure, resulting in up to 36% higher hardness and notable increases in modulus and strength.

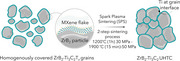

## 1 Introduction

Ultra‐high temperature ceramics (UHTCs) have been a subject of extensive research since the 1960s, with renewed interest in developing materials for hypersonic vehicles, atmospheric re‐entry systems, and other extreme environment applications emerging in the 2000s.^[^ ^1^, ^2^ ^]^ The most studied UHTCs are hafnium diboride (HfB_2_), tantalum carbide (TaC), and zirconium diboride (ZrB_2_), each offering unique advantages. Among UHTCs, zirconium diboride (ZrB₂) stands out due to its exceptional properties, including high melting point (>3200 °C), high strength at elevated temperatures (up to 800 MPa at 1600 °C), excellent thermal conductivity, and ablation resistance.^[^ ^3^, ^4^, ^5^, ^6^ ^]^ These characteristics make ZrB₂ a promising candidate for use in thermal protection systems, rocket nozzle inserts, sharp leading edges of hypersonic vehicles, and other components that must withstand extreme thermal and mechanical stresses.^[^ ^7^, ^8^, ^9^, ^10^, ^11^, ^12^, ^13^, ^14^ ^]^

However, monolithic ZrB₂ ceramics suffer from inherent brittleness and poor sinterability due to their strong covalent bonding and low self‐diffusion coefficients, making it challenging to achieve full densification without applying very high temperatures and pressures.^[^ ^15^ ^]^ Additionally, ZrB₂ is prone to oxidation at elevated temperatures, which can limit its performance in oxidative atmospheres.^[^ ^16^ ^]^ To address these shortcomings, various strategies have been employed, including the incorporation of secondary phases such as transition metals, oxides, carbides, silicides, and nitrides.^[^ ^15^, ^17^, ^18^ ^]^ These additions have been shown to enhance oxidation resistance and improve mechanical properties by promoting densification and inhibiting grain growth.

In recent years, the addition of 1D and 2D materials, such as carbon nanotubes (CNTs), graphene, and graphene oxide, has been explored to improve the sinterability and thermomechanical performance of UHTCs.^[^ ^19^, ^20^, ^21^, ^22^, ^23^, ^24^, ^25^ ^]^ These nanomaterials act as sintering aids and reinforcements, promoting densification and enhancing properties such as fracture toughness, hardness, and strength through mechanisms, including grain boundary strengthening, crack deflection, and pull‐out effects.^[^ ^24^ ^]^ However, challenges remain in achieving uniform dispersion, strong interfacial bonding, and thermal stability of these additives at the high processing temperatures required for UHTCs.

Compared to other 1D and 2D additives, MXenes are 2D layered transition metal carbides, nitrides, or carbonitrides,^[^ ^26^ ^]^ with similar compositions to some of the carbide UHTCs, such as TiC and TaC.^[^ ^15^ ^]^ MXenes are denoted by the chemical formula M*_n+1_* X*_n_* T*_x_*, where M represents a transition metal from groups 3 to 6 of the *d*‐block elements, X represents the non‐metal (C and N), and T*_x_* represents diverse surface functional groups on the MXenes basal planes, such as –O, –F, –OH, and –Cl and other halogens.^[^ ^27^, ^28^ ^]^ In MXenes, the *n*+1 atomic planes of M are arranged with interleaving X atoms (*n* layers), in a M‐X‐M‐X type arrangement. For example, Ti_3_C_2_T*_x_* MXene is atomically layered with alternating planes of Ti‐C‐Ti‐C‐Ti with surface terminations attached to the outer Ti layers.

MXenes are derived from their layered precursor phases, usually MAX phases, which are ternary or quaternary transition metal carbides;^[^ ^29^ ^]^ where M and X represent the corresponding transition metal and the non‐metal components similar to MXenes, and A represents a layer of atoms typically from groups 13 to 15 of the periodic table. Selective etching of the A layers from the MAX phase results in their 2D MXene counterparts. MXenes inherit the chemical composition, crystal structure, morphologies, and some of the physical properties of their corresponding precursor MAX phases.^[^ ^30^, ^31^ ^]^

The titanium carbide MXene (Ti_3_C_2_T*_x_*) is extensively studied within the MXene family,^[^ ^30^ ^]^ offering outstanding mechanical properties among all solution‐processed 2D materials (≈480 GPa stiffness for single flake),^[^ ^32^, ^33^ ^]^ high electrical conductivity (up to 24 000 S cm^−1^ in film form),^[^ ^34^ ^]^ excellent colloidal stability (zeta potential of≈−40 mV in water at pH 7) and adaptability in a wide range of protic and aprotic solvents.^[^ ^35^ ^]^ These attributes have enabled its implementation in many applications, including energy storage, catalysis, electronics, and biomedical fields.^[^ ^36^, ^37^, ^38^ ^]^ The Ti_3_C_2_T*_x_* system is particularly well‐known for its use in charge storage, electromagnetic interference (EMI) shielding, and catalysis, owing to its large aspect ratio, layered morphology, high electrical conductivity, and chemically active basal planes.^[^ ^39^, ^40^ ^]^ Recent studies have also explored its electrical properties for developing porous MXene‐ceramic architectures for multifunctional applications.^[^ ^41^ ^]^

However, the use of MXene in structural and mechanical applications remains relatively unexplored. Only a limited number of studies have examined its mechanical properties, tribological performance, and potential as a reinforcing additive in metal and ceramic composites. These studies have primarily focused on evaluating its hardness, toughness, and wear resistance, as well as its ability to improve the strength, durability, and frictional characteristics of composites when integrated into different matrices.^[^ ^42^, ^43^, ^44^, ^45^, ^46^, ^47^, ^48^, ^49^, ^50^, ^51^ ^]^ In most composite processing approaches, materials incorporating MXene as an additive must be sintered at high temperatures—above 500 °C for early transition metals and above 1000 °C for ceramics. Our previous work demonstrated that Ti_3_C_2_T*_x_* undergoes a phase transformation from a hexagonal structure to cubic vacancy carbide (TiC*_y_*) upon annealing in controlled environments above 1200 °C while retaining its lamellar structure and morphology.^[^ ^52^ ^]^

Carbon vacancy transition metal carbides, particularly in bulk 3D structures, are known to exhibit unique structure‐property relationships, including anomalous hardening behavior attributed to microstructural effects and dynamic elastic constants.^[^ ^53^ ^]^ Moreover, cubic TiC is classified as a UHTC, with a high melting point (≈3160 °C), superior hardness, and excellent wear resistance, making it suitable for extreme environment applications.^[^ ^54^, ^55^ ^]^ While some studies have reported on interactions between MAX phases (MXene precursors) and UHTCs,^[^ ^56^, ^57^ ^]^ MXenes have not yet been extensively investigated in conjunction with diboride‐based UHTCs. To our knowledge, only one prior study has explored MXene's role in carbide‐based UHTCs, specifically TiC.^[^ ^47^ ^]^ In that study, the phase transition of Ti_3_C_2_T*_x_* to TiC enhanced the bending strength of the composite by ≈15% and improved the indentation toughness by ≈50% due to strong interfacial bonding.

In this work, we investigate the potential of MXene as a 2D additive for high‐temperature applications with a conventional diboride UHTC, ZrB_2_. Our approach involves the use of 2D carbide MXenes to conformally encapsulate 3D UHTCs. This strategy leverages the unique properties of 2D MXenes to create interfaces and grain boundaries that are inherently compatible with the UHTC matrix. By doing so, we believe MXene can aid in enhancing the mechanical and thermal properties of the composite material while maintaining the integrity and performance required for extreme environment applications.

**Figure 1:**
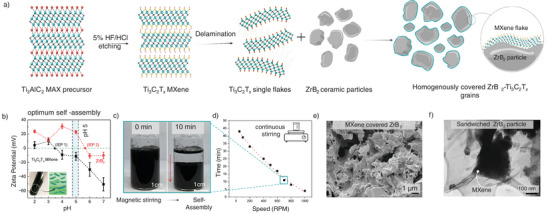
a) Schematic of MXene synthesis from the precursor MAX phase Ti_3_AlC_2_ using a mild 5% HF/HCl etching route. The etched MXene was delaminated using 0.02 mol LiCl solution (0.475 mol). Single flake MXene suspensions were balanced to 5 pH. The ceramic slurries of ZrB_2_ in water (0.2g mL^−1^) also 5 pH balanced were added to the MXene suspension with continuous stirring. MXene flakes coated on the ZrB_2_ particles were obtained due to gravity assisted self‐assembly process; b) Zeta‐potentials versus pH of the MXene (flake size: 2–3 µm) and ZrB_2_ (particle size: 750 nm) indicating pH 5 as optimum environment for self‐assembly. The concentrations of the test samples were 0.05 mg mL^−1^. c) photo of MXene‐ZrB_2_ mixed slurries at time (t) = 0 min and 10 min after mixing. The complete separation of the solvent (water) and the self‐assembled green bodies was observed due to self‐assembly of the MXene flakes with ZrB_2_ particles; d) the average settling time (time required for complete self‐assembly) as a function of stirring speeds. Increasing the speed of mixing decreases the self‐assembly times; e) Scanning electron microscopy (SEM) of 2.5 wt.% MXene‐ZrB_2_ green bodies, f) bright‐field TEM micrograph showing a ZrB_2_ grain wrapped with MXene flakes.

To achieve this, we used the negative zeta potential of MXene and the positive zeta potential of ZrB_2_ in acidic pH and developed a self‐assembly‐driven green body synthesis method to conformally cover the 3D UHTC grains (ZrB_2_) with 2D sheets of Ti_3_C_2_T*_x_* MXene (**Figure** **1**a) at different weight fractions.^[^ ^58^ ^]^ Following the preparation of homogenous green bodies, we sintered the MXene‐wrapped ZrB_2_ powders via spark plasma sintering (SPS) by applying 50 MPa pressure at 1900 °C. The addition of MXene as low as 0.7 vol.% (0.5 wt.%) into ZrB_2_ showed improvement in densification from ≈88% in pure ZrB_2_ to ≈96% for ZrB_2_ with MXene after the SPS. Additionally, our results indicate that MXene incorporation reduces the inherent oxygen content in commercially available ZrB_2_ upon sintering.

Furthermore, we provide evidence of diffusion and solid solution formation at the MXene‐ZrB₂ interfaces, which likely contribute to the enhanced densification and the evolution of a core–shell microstructure. We observed the emergence of numerous dislocations and their polygonization at grain interfaces,^[^ ^59^, ^60^ ^]^ marking the first observation of core–shell microstructure formation in a 2D MXene–3D ceramic system. This structural evolution suggests the potential for high strength retention at elevated temperatures, further highlighting MXene's viability as a reinforcing additive in UHTCs for extreme environment applications.

This study demonstrates two key findings that can enable MXenes’ implementation as additive materials in UHTCs: a) a scalable, aqueous, electrostatically driven self‐assembly process for preparing green bodies of nano‐flake carbide additives into ZrB_2_ without the need for surfactants or stabilizing agents. We anticipate that this method is also applicable to other MXenes and UHTCs (such as HfB_2_ and Ta_4_C_3_T*_x_*). b) We provide evidence that MXenes enable the production of nearly fully dense ceramics with enhanced mechanical properties, unique microstructure, and strengthening mechanisms for their high‐temperature applications.

## 2 Results and Discussion

### 2.1 Synthesis and Characterization of Ceramic Green Bodies

We prepared homogeneous green bodies of MXene‐ZrB_2_ using electrostatic interactions in aqueous solutions by controlling the pH, to achieve the desired zeta potentials for successful self‐assembly. The negatively charged surface terminations of MXenes create a balance between the repulsive coulombic forces of the double layer and the attractive forces, resulting in electrostatic stabilization of the flakes in aqueous media.^[^ ^61^ ^]^ Coupled with the positive zeta potentials of ZrB_2_ in acidic pH, we can harness the charge mismatch to drive electrostatic self‐assembly between the ZrB_2_ particles and the MXene flakes.

Figure 1b shows the zeta potential versus pH for ZrB_2_ slurries (in water) and Ti_3_C_2_T*_x_* MXene (in water) for a pH range of 2–7. At neutral pH, the measured zeta potential of our Ti_3_C_2_T*_x_*, with average flake sizes of 2 ± 0.33 µm, was highly negative at −50 ± 7 mV, which agrees well with previous reports on MXene colloidal suspension stabilities.^[^ ^35^ ^]^ The highly negative zeta potential of MXene at neutral pH results in aqueous dispersions of Ti_3_C_2_T*_x_* MXene that are stable with no surfactant assistance. By altering the pH from 7 to pH 2, the surface zeta potential becomes less negative, with the iso‐electric point of MXene (IEP_1_) being ≈ pH 4.3. Visible flocculation is observed in MXene suspension as pH decreases below 4.

The ZrB_2_ powder with particle sizes of 750 nm, on the other hand, exhibits a positive surface charge at pH 5 (+24 ± 2.6 mV) with the IEP_2_ estimated around pH 5.7, which is close to the previously reported values.^[^ ^62^, ^63^ ^]^ The slight changes in values could be due to the particle size variations. For example, typical IEPs were observed around pH 4.7 for ZrB_2_ with particle sizes of 2 µm.^[^ ^64^ ^]^ ZrB_2_ suspensions were highly unstable at neutral pH (−10 ± 3.8 mV) with greater stabilities with an increase in acidity of the solvent.

The optimum electrostatic charge‐driven self‐assembly between the 3D ZrB_2_ ceramic grains and 2D Ti_3_C_2_T*_x_* MXene sheets is obtained around pH 5 at room temperature with visible phase separation between the solute and the solvent phases (Figure 1c). We tested different mixing speeds (stir plate at 100–1000 RPM) after adding the ZrB_2_ slurries into the MXene solution at pH 5. A trend between time for phase segregation versus mixing speeds was observed, with longer separation times required for lesser velocities of mixing (Figure 1d). We selected 700 rpm as the stirring speed with a phase separation time of 10 min for all our samples (turbid solvent to clear transparent solvent‐Figure 1c) (Video S1, Supporting Information). A detailed experimental protocol is discussed in the methods section of this study. We note here that the time required for self‐assemblies is size‐dependent, with a shorter settling time required for larger particle sizes (Figure S1, Supporting Information). This indicates that in addition to the electrostatic self‐assembly phenomenon, the weight of the ceramic particles plays a role in the separation process, and most probably, this gravity separation can limit the electrostatic self‐assembly. We selected ZrB_2_ with ≈ 750 nm average particle size to minimize the effect of gravity in our self‐assembly process.

We prepared homogeneous green bodies of MXene‐ZrB_2_ with MXene loadings of 0.5 wt.%, 1.5 wt.%, 2.5 wt.%, 5 wt.%, 10 wt.%, and 15 wt.%. Figure 1e is an SEM micrograph of a 2.5 wt.% MXene‐ZrB_2_ self‐assembled green‐body powders showing coverage of the diboride grains with MXene sheets (see Figure S2, Supporting Information for comparison). We estimated that as low as 0.5 wt.% (0.7 vol.%) of MXene is ample to sufficiently cover the ceramic grains in their entirety. The geometrically derived calculations and the SEM micrographs of the green bodies with varying MXene wt.% loadings are shown in Figure S2 (Supporting Information). The presence of MXenes is differentiated by the wrinkled nature of the flakes on rigid ZrB_2_ particles, with increase in MXene loadings increasing the smoothness of the otherwise rugged boride particle as seen in Figure S2a (Supporting Information). An increase in the number of stacks or layers of MXene sheets is seen with the increase in wt.% of the MXene loadings (Figure S2a–f, Supporting Information). The transmission electron microscopy (TEM) image of self‐assembled green bodies with 2.5 wt.% MXene (Figure 1f) shows an example of ZrB_2_ grains wrapped with MXene flakes. The contrasting dark‐field and the bright‐field TEM images (Figure S3a, Supporting Information) show a non‐transparent ZrB_2_ grain with an electron translucent MXene flake covering the grain. The phases are differentiated by the wrinkles and reflections on the MXene flakes due to conformal coverage on top of the ceramic grains. Single flake MXene is shown in Figure S3b (Supporting Information) TEM micrograph.

To better understand the effect of gravity‐assembly versus self‐assembly, we used multi‐layer MXene (non‐delaminated) to prepare green bodies via a similar mixing process. Unlike the single‐to‐few layers delaminated MXene that readily (within minutes) self‐assembled with ZrB_2_, the multi‐layer particles of MXene did not exhibit any self‐assembly behavior (Figure S4a, Supporting Information). By increasing the wait time post‐mixing to more than 1 h, a clear solution was obtained for the multi‐layer MXene‐ZrB_2_ mixtures. However, SEM analysis did not show any self‐assembly (Figure S4b,c, Supporting Information).

Another variable that we investigated in self‐assembly effectiveness is the aging of MXene. We observed that a week‐old MXene suspension (stored in plastic vials in a dark drawer at ambient conditions) has ≈30% less negative zeta potential (≈−28 mV at neutral pH for a week‐old suspension as opposed to ≈−50 mV for freshly synthesized 1‐day old MXene). This is primarily attributed to the active hydrolysis and formation of titanium oxide nanograins on MXene that disrupt the surface charges, leading to unstable suspensions.^[^ ^65^ ^]^ To avoid any variation in our green body preparation, we used freshly synthesized MXene solutions (within an hour of the MXene delamination) for self‐assembly.

### 2.2 X‐Ray Diffraction Studies on the Green Bodies

**Figure 2:**
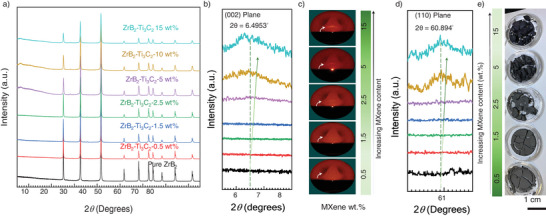
a) XRD patterns for the green bodies with variable MXene content. b) The out‐of‐plane peak at 6.49° (002) of MXene is seen with increasing intensities with an increase in MXene content. No peak is observed up to 2.5 wt.% addition indicating a high order of conformity with the matrix. The 5, 10, and 15 wt.% samples show an emerging (002) peak indicating that the MXene flakes have stacked on the surface of the matrix. c) 2D X‐Ray diffractograms (2θ = 5–10°) showing the emergence of the out‐of‐plane peak (002) with an increase in MXene loading. d) The XRD in‐plane peak (110) of MXene with increase in intensity with increasing the MXene loadings, e) Photographs of MXene‐ZrB_2_ dried powder mixtures showing the green bodies darkening with increasing MXene loadings (light grey to black).

The green bodies of ZrB_2_ mixed with variable MXene loadings were characterized by 2D X‐Ray diffraction (XRD) (**Figure** **2**a). We had previously reported that the 2D XRD method is a reliable tool to characterize a MXene‐metal composite containing a small volume fraction of MXene loadings with a greater degree of detection accuracy.^[^ ^66^ ^]^ Using similar characterization principles, we observed that the MXene‐ZrB_2_ green bodies do not show any characteristic MXene peak at 2θ = 6.5^°^ to 7.5^°^ where a diffraction signal corresponding to the (002) plane of MXene is typically seen, for green‐bodies with 0.5 wt.% (red), 1.5 wt.% (blue), and 2.5 wt.% (green) weight loadings (Figure 2b). A signal response at 6.49^°^ is seen for green bodies containing at least 5 wt.% loadings and this intensity increases for the 10 and 15 wt.% MXene‐ZrB_2_ green bodies, respectively.

The peaks shown in Figure 2b are created by the integration of the diffraction cones that we captured with the 2D detector (Figure 2c). In these diffraction cones, we also can see the emergence of 2θ = 6.49^°^ by increasing the MXene content. Further, we observed the appearance of the in‐plane (110) MXene peak at 60.9^°^ 2θ and an increase in its intensity by increasing MXene content (Figure 2d). SEM micrographs show ZrB_2_ grain coverage at 0.5, 1.5, and 2.5 wt.% MXene. However, the lack of diffraction signal in the 2θ range of 5–10^°^ with the 2D wide angle detector suggests effective conformal coverage of the ZrB_2_ grains with MXene flakes with minimum restacking of the MXene flakes in the 0.5 wt.% and up to 2.5 wt.% MXene samples. Conversely, relatively sharper peaks with higher MXene loadings (5 wt.% and higher) indicate stacking of MXenes that lead to detectable diffraction responses (Figure 2d). We have also observed that the green bodies are visibly darker in color (light gray for 0.5 wt.% MXene to black for 15 wt.% MXene) as shown in Figure 2e. The 15 wt.% MXene‐ZrB_2_ green bodies were also lustrous (Figure 2e), which is normally observed for delaminated multi‐layer MXene clays in which there is considerable stacking of the MXene flakes.

### 2.3 Field‐Assisted (Spark Plasma) Sintering (SPS) of the Green Bodies

**Figure 3:**
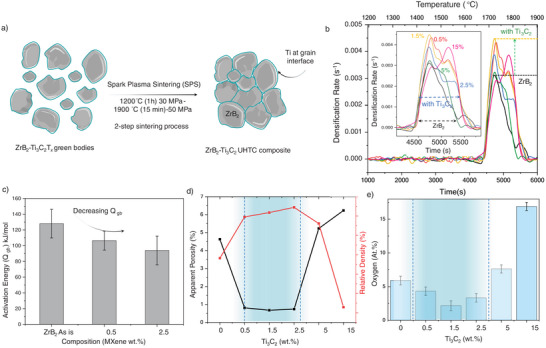
a) Schematic of the ZrB_2_‐MXene green bodies before and after SPS sintering. The phase transformed MXene into TiC*_y_* during sintering is shown in green, b) Densification rates (s^−1^) versus time (s) and temperature (°C) plots for pure ZrB_2_, and ZrB_2_‐MXene composites. An increase in net densification rates is seen for samples with MXene addition. The magnified densification window also shows an increment in densification windows of ZrB_2_‐MXene (≈1000 s) compared to pure ZrB_2_ (≈750 s), c) The activation energies (Q_gb_) versus composition, pure ZrB_2_, ZrB_2_‐MXene (0.5 wt.%), ZrB_2_‐MXene (2.5 wt.%). The activation energies were estimated from the ram displacement data during the sintering process. A decreasing trend in the grain boundary activation energies is observed with an increase in MXene weight loading above 5 wt.%. The range of activation energies indicates a dominant grain boundary diffusion mechanism that promotes sintering in this system. d) Apparent porosity and relative densities (right y‐axis) of sintered composites versus composition. The porosity decreases and the relative density increases with the addition of MXene up to 2.5 wt.% MXene addition. A decrement in relative densities is observed for samples with 5 and 15 wt.% MXene‐ZrB_2_ samples. e) Oxygen at.% as measured by EDS versus composition bar chart showing an increase in oxygen content above a threshold (2.5 wt.% MXene addition). The bar chart is normalized against the control sample (0 wt.%).

Next, to optimize the densification and microstructural properties of our ZrB₂‐MXene composites, we conducted an extensive investigation of multiple sintering protocols and variables. The green bodies were sintered using SPS under inert atmospheres,^[^ ^67^ ^]^ as schematically shown in **Figure** **3**a. Sintering temperatures ranging from 1000  to 1900 °C were systematically evaluated to understand their effects on sintering behavior and microstructure evolution. We tested different sintering cycles to achieve densification, namely: single‐step sintering: a direct ramp to 1900 °C, and two‐step sintering: incorporating a hold at 1200 °C before ramping to 1900 °C. This approach allowed us to identify the optimal conditions for achieving high relative densities with minimal porosity. The SEM micrographs and EDS scans of samples sintered samples via different sintering settings are shown in Figure S5 (Supporting Information).

A dwell temperature of 1200 °C was added based on early reports^[^ ^68^, ^69^, ^70^ ^]^ where complete removal of surface functional groups in MXenes is expected beyond 800 °C up to 1200 °C, to enable removal of oxygen terminations from the MXenes surfaces in inert environments. Further, studies on ZrB_2_ have shown that the initiation of densification for ZrB_2_ systems is predominantly between 1600 and 1730 °C,^[^ ^71^, ^72^ ^]^ and the densification regimes are dependent on the heating rate, impurities, and particle size distribution. Additionally, the presence of sintering additives decreases the densification onset temperatures where consolidation is expected due to additive‐driven inter‐phase interactions.^[^ ^12^, ^15^, ^73^ ^]^

Based on these known conditions (Figure S6a, Supporting Information), we chose our sintering condition as a two‐step sintering process where the sample was soaked under uniaxial pressure of 30 MPa at 1200 °C for 1 h to enable the removal of surface functional groups, followed by a second temperature dwell at 1900 °C with a uniaxial pressure of 50 MPa and dwell time of 15 min to densify the green bodies. The sintering cycle conditions (temperature and pressure), and the ram displacement rates are shown in Figure S6b,c (Supporting Information). The densification rates were measured from real‐time ram displacement monitoring during sintering. The empirical relations are provided in Text S6 (Supporting Information).

Figure 3b shows the densification rate (s^−1^) versus time (0–100 min) of sintering. We observed enhanced densification behavior in samples with MXene, compared to the pure ZrB_2_ powders which were consolidated via an identical sintering cycle. An increment in ram displacement above 1650 °C for the pure ZrB_2_ as well as MXene‐ZrB_2_ samples is observed (Figure S6c, Supporting Information). The average displacement was increased for samples with 0.5, 1.5, 2.5, and 5 wt.% MXene compared with the pure ZrB_2_ sample with the highest for ZrB_2_‐1.5 wt.% MXene. In addition, the densification window increased for samples with MXene content (Figure 3b‐inset). The increase in instantaneous densities (%) versus temperature for pure ZrB_2_ and 0.5 wt.% MXene‐ZrB_2_ are shown in Figure S6d (Supporting Information). The presence of MXene in the ZrB₂ matrix is likely to promote enhanced particle rearrangement during the initial stages of sintering. MXenes can act as a lubricant within the powder matrix, reducing friction between particles. This lubricating effect facilitates particle rearrangement under applied pressure, allowing for better packing and higher green densities at lower temperatures. Similar phenomena have been reported in other composite systems, such as graphene nanoplatelet‐reinforced tantalum carbide, where the two‐dimensional nature of the reinforcement aids in achieving higher initial densification rates,^[^ ^24^ ^]^ and MXene‐alumina composites, where the lubricating nature of MXene along with creep and grain boundary sliding, were attributed causes for increased densification.^[^ ^74^ ^]^ Further, the layered structure of MXenes can serve as preferential sites for grain boundary sliding. This effect becomes particularly significant at higher temperatures (above 1650 °C), where the enhanced mobility of atoms promotes sliding at the grain boundaries. The presence of MXene can act as a facilitator for this process, allowing for more efficient grain boundary diffusion and sliding, thereby reducing the activation energy required for densification.

To further understand the interface interactions and the dominant mechanism influencing densification, we estimated the activation energies of the composites (Figure 3c). We assumed that the instantaneous densification rate can be equated to an Arrhenius equation relating activation energies, and densification rates using Coble's equation for estimating boundary diffusion‐controlled creep.^[^ ^75^ ^]^ The Arrhenius plots and derivations and provided in Figures S7 and Text S7 (Supporting Information). The estimated range of activation energies for pure ZrB_2_ (110–150 kJ mol^−1^) and ZrB_2_‐MXene‐2.5 wt.% (80–120 kJ mol^−1^) indicate grain boundary diffusion as a dominant diffusion mechanism.^[^ ^76^ ^]^ A nominal decrease in activation energies compared to pure ZrB_2_ suggests faster diffusion rates due to increased MXene presence at higher MXene wt.%. This is facilitated by larger aspect ratios and greater surface areas, with nanometer‐thick and laterally micron‐sized 2D MXene flakes wrapping the ZrB_2_ grains, thereby providing greater diffusion paths. Additionally, MXene's lower phase transformation temperature, owing to its nanometer‐thick size further enhances diffusion, as previous studies have indicated that atom diffusion in MXene flakes can initiate at temperatures below 1000 °C.^[^ ^77^, ^78^ ^]^

We measured the relative densities and porosities of the sintered specimens using Archimedes’ principle. Figure 3d illustrates the variation in relative densities (%) and apparent porosities (%) as a function of MXene content (wt.%). The relative density increases from ≈89% for pure ZrB₂ to ≈94% for 0.5 wt.% MXene‐ZrB₂, with a corresponding decrease in porosity from 4.5% to 0.9%. This trend continues up to 2.5 wt.% MXene addition (≈95% relative density). However, beyond 2.5 wt.%, a decline in relative density is observed, with samples containing 5 and 15 wt.% MXene exhibiting lower densification. The initial increase in densification can be attributed to MXene's role as a sintering aid, promoting particle rearrangement and grain boundary mobility and enhanced interfacial bonding and diffusion during sintering.

However, at higher MXene loadings (≥5 wt.%), the re‐stacking of MXene flakes leads to the formation of multilayer structures, which hinder effective densification. Trapped water within these multilayers can contribute to MXene oxidation at relatively low temperatures (below 600 °C), resulting in secondary phase formation that disrupts the sintering process. Additionally, partial oxidation of ZrB_2_ can introduce further porosity.^[^ ^79^ ^]^ To analyze porosity variations, a machine learning‐based image segmentation approach was applied to SEM micrographs, as described in the methods section. The analysis confirms that while low MXene loadings reduce porosity, excessive MXene incorporation increases it due to re‐stacking and oxidation effects.

### 2.4 Characterization of MXene‐ZrB_2_ UHTCs

We analyzed the sintered samples for elemental composition and estimated the net oxygen content using energy‐dispersive X‐ray spectroscopy (EDS). EDS analysis (Figure 3e) shows that the oxygen at.% in monolithic (pure) ZrB_2_ is ≈5 ± 1.3 at.%., which indicates the as‐received powder contained oxygen and remained in the final sample after sintering. However, the addition of MXene into ZrB_2_ (at 0.5, 1.5, and 2.5 wt.% MXene) reduced the oxygen content, with the lowest being 2.5 at.% oxygen for the 1.5 wt.% Ti_3_C_2_T*_x_*‐ZrB_2_ sample (Figure 3e). This suggests that MXene can act as a reducing species during sintering, thereby lowering the final oxygen content in the specimen. Considering that every 1‐nm‐thick Ti_3_C_2_T*_x_* MXene flake has oxygen terminations (for example, Ti_3_C_2_(OH)_0.12_O_0.54_ and Ti_3_C_2_O_0.254_OH_0.456_F_1.218_Cl_0.072_ for HF‐HCl etching route),^[^ ^80^ ^]^ one can argue that MXene can only lead to an increase in the oxygen content of the ceramic and even oxidize it. However, it is known that the surface terminations can be removed at temperatures between 800 and 1200 °C.^[^ ^68^, ^69^ ^]^ This rationale led us to select a two‐step SPS sintering process with a 1‐hour dwell at 1200 °C. The observed decrease of oxygen in ZrB_2_ upon the addition of up to 2.5 wt.% Ti_3_C_2_T*_x_* MXene indicates the successful removal of surface terminations.

In addition to surface terminations, we must also consider trapped moisture and water molecules between the MXene flakes. While the usual practice for drying MXene flakes is to vacuum anneal them at ≈ 200 °C to remove water, a recent study has indicated that trapped water molecule removal is dependent on the number of stacked MXene flakes.^[^ ^79^ ^]^ The water trapped between the stacked MXene flakes requires more energy and a higher annealing temperature than water removal from MXene single flakes. If not removed, this trapped water can lead to the formation of nano‐sized TiO_2_ during higher temperature annealing (> 400 °C) in stacked MXenes.^[^ ^79^ ^]^ The entrapment of water and subsequent oxide formation can explain the increase in the oxygen content for samples containing 5 wt.% and 15 wt.% MXene, with estimated oxygen content of 5–7 at.% and >15 at.%, respectively. As discussed earlier, 0.5 wt.% of MXene is needed for a single flake coverage of the ZrB_2_ grains and 1.5 wt.% can lead to few‐layer stacking. Consequently, increasing the MXene addition beyond 1.5 wt.%, leads to restacking of multiple MXene flakes and the trapped moisture between these flakes can lead to some oxidation and an increase in net oxygen content after sintering.

Another source of oxygen could be the oxygen atoms in the carbon sites (carbon sub‐lattice) of Ti_3_C_2_T*_x_*, which originated from its Ti_3_AlC_2_ precursors. It is known that all Ti_3_C_2_T*_x_* made to date are oxycarbides (Ti_3_(C,O)_2_T*_x_*).^[^ ^80^ ^]^ To understand the effect of this oxygen source in the sintered ceramic, we prepared Ti_3_C_2_T*_x_* MXene derived from a modified Ti_3_AlC_2_ (with the use of excess metal), which is known to contain near 0 at.% oxygen in the structure (sub‐lattice).^[^ ^80^ ^]^ We labeled this MXene as optimized‐MXene and its synthesis protocol is provided elsewhere.^[^ ^81^ ^]^ Using the optimized‐MXene, we prepared a set of ZrB_2_‐optimized‐MXene samples following the same sintering process as for ZrB_2_‐MXene. We observed a similar decremental trend in the net oxygen content by increasing optimized‐MXene content up to 1.5 wt.%. The total oxygen content in the sintered ZrB_2_‐2.5 wt.% optimized Ti_3_C_2_T*_x_* sample was 2.1 ± 0.9 at.%, which is similar to the O content of the regular MXene samples shown in Figure 3e. The overall observation here leads us to believe that the addition of optimized wt% of Ti_3_C_2_T*_x_* MXene (up to 2.5 wt.% here) acts as a reducing agent during sintering and prevents the formation of oxides in the composite. However, the addition of large amounts of MXene (> 5 wt.%) leads to 2D flake stacking, which can cause oxidation mainly due to water molecules’ entrapment. Further discussion on the formation of oxides and the role of MXenes is presented in Text S8 and Figure S8 (Supporting Information).

We also performed EDS mapping on fractured samples with and without MXene addition shown in Figure S9 (Supporting Information). We note that the net oxygen content in samples with MXene was observed to be ≈2 at.% compared to ≈5.9 at.% in pure ZrB_2_ samples. The distributions of Ti and C were uniform across the micrograph with no distinction or agglomeration in Ti was observed (Figure S9, Supporting Information).

**Figure 4:**
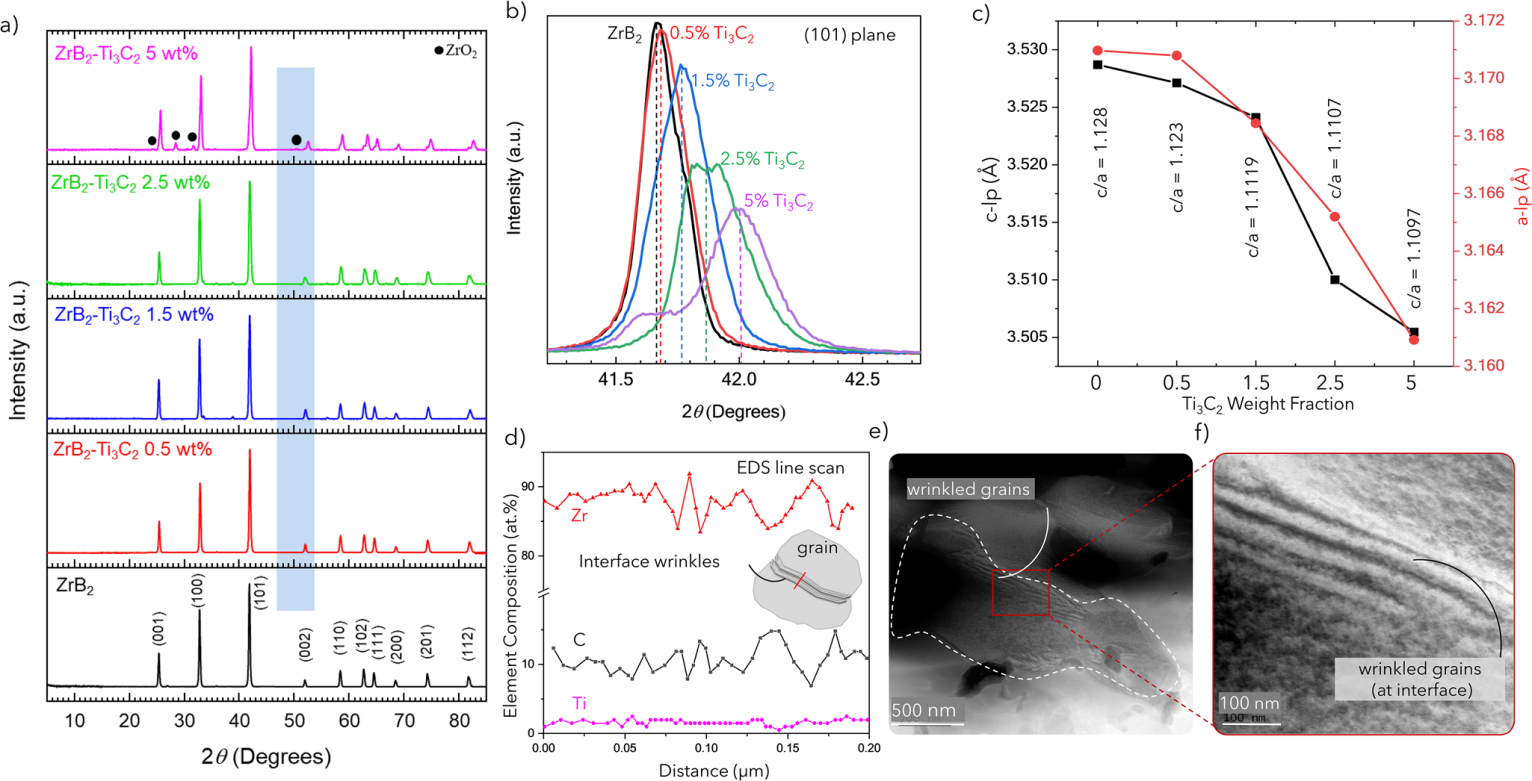
a) XRD patterns of spark plasma sintered MXene‐ZrB_2_ composites with variable MXene loadings (0.5 wt% to 5 wt%), presence of ZrO_2_ in detectable quantities is seen in samples with 5 wt.% MXene. b) peak shift in (101) plane of the composite with variable MXene loadings. The systematic right shift in the peak indicates shrinkage of lattice which suggests diffusion of species. Further, peak broadening and splitting of shoulder on (101) plane in the 2.5 wt.% and 5 wt.% sample shows formation of a secondary solid solution phase. c) The c‐lattice parameter and a‐lattice parameters versus MXene wt.% loading for the sintered samples. A decrease in the lattice parameters (decrease in the c/a ratios) indicates formation of a (Zr,Ti)B_2_ phase with increase in MXene concentration in the composite. d) EDS line scan of the wrinkled grain showing a mixed composition of (Zr, Ti)B_2,_ e, f) TEM micrographs of a 1.5 wt.% MXene‐ZrB_2_ sintered composite showing wrinkling at the grain boundaries as a possible reminiscence of the 2D MXene.

After EDS analysis and estimation of elemental fractions, we investigated the sintered MXene‐ZrB_2_ samples via XRD to identify the constituent phases (**Figure** **4**a). The high‐intensity peaks belong to ZrB_2_ (labeled in Figure 4a bottom), and the most intense peak is observed at 2θ ≈41.67° for the ZrB_2_ (101) plane. The XRD results did not show any peaks for oxide formations (TiO_2_ or ZrO_2_) for samples with 0.5, 1.5, and 2.5 wt.% MXene. Small peaks of ZrO_2_ are seen in the 5 wt.% MXene‐ZrB_2_ samples. Samples with 2.5 and 5 wt.% MXene also shows small peaks corresponding to the (111) peak of ZrC at 2θ = 32.8°, indicated by an increase in shoulder intensities of the (100) plane of ZrB_2_ at 2θ = 33.1°. Further, sintered ZrB_2_ samples with 15 wt.% MXene showed several ZrO_2_ peaks as well as ZrC, TiC_y_, and (Zr,Ti)B_2_ solid solution. We did not observe any TiO_2_ peaks in these samples either (Figure S10, Supporting Information).

The presence of ZrO_2_ in samples with 5 wt.% MXene is likely due to the stacking of MXene multilayer flakes, which increased the overall trapped water in the composite and reacted with ZrB_2_ during sintering, leading to the formation of ZrO_2_. It is important to note that the formation of ZrO_2_ was only observed in small amounts in the 5 wt% MXene‐ZrB_2_ samples, indicating that the oxide formation process was limited. The absence of ZrO_2_ peaks in samples with lower MXene content (0.5, 1.5, 2.5, 2.5 wt.%) suggests that there is a threshold concentration of MXene above which the oxidation of ZrB_2_ becomes more pronounced.

After identifying the main phases in the samples, we focused on analyzing ZrB_2_ peak evolution by the addition of MXene. The XRD analysis indicated a notable right‐shift of the most prominent (101) peak from 41.67° (pure ZrB_2_) to 41.7° (0.5 wt.%), 41.75° (1.5 wt.%), 41.7° (2.5 wt.%), and 42° (5 wt.%) accompanied by peak broadening. The (101) peak for the 5 wt.% sample also exhibited a splitting at 41.7° (Figure 4b). Similar peak shifts were observed for all the peaks, with higher 2θ peaks exhibiting greater shifts. We estimated the lattice parameters (LPs) for all sintered samples using the Neilson‐Riley function (Figure 4c). A simultaneous decrease in the *a*‐lattice parameters (*a*‐LPs) and the *c*‐lattice parameters (*c*‐LPs) was observed, with pure ZrB_2_ exhibiting *a*‐LP = 3.17097 ± 0.0013 Å and *c*‐LP = 3.5287 ± 0.0009 Å, while the 5 wt.% MXene‐ZrB_2_ sintered sample exhibited an *a*‐LP = 3.15977 ± 0.0011 Å and *c*‐LP = 3.50463 ± 0.0014 Å. The lattice constants are provided in Table S1 (Supporting Information). Additionally, there was an increase in the calculated full width at half maximum (FWHM), indicating the development of strains in the lattice (Figure S11, Supporting Information). The high symmetry of the diffraction peaks further suggests the evolution of a congruent single phase in the system.

In Figure 4c we present the *c*/*a* ratios for each sample, which shows a decreasing trend from *c*/*a* = 1.128 for the pure ZrB_2_ sample to *c*/*a* = 1.1096 for ZrB_2_‐2.5 wt.% MXene. Estimations for pure ZrB_2_ show *c*/*a* ratio of 1.1142 while the c/a ratio of the solid‐solution phase with 1:1 metal composition (Zr_0.5_Ti_0.5_B_2_) is calculated to be in the range 1.0962–1.1057.^[^ ^82^ ^]^ These results indicate shrinkage of lattice interplanar spacing upon MXene addition, due to the formation of (Zr_1‐x_Ti_x_)B_2_ solid solution at the ZrB_2_‐Ti_3_C_2_T*_x_* interfaces in these UHTCs. Moreover, the splitting of peaks in XRD is also indicative of a core–shell microstructure which we have discussed in detail in the following sections.^[^ ^83^ ^]^

To confirm the diffusion of Ti and formation of (Zr_1‐*x*_Ti_x_)B_2_ solid solution, we examined the interfaces using transmission electron microscopy (TEM) on thin, mechanically milled samples of sintered ZrB_2_‐1.5 wt.% MXene sample. EDS line scans were performed to estimate the composition across grains (Figure 4d) showing the presence of Zr, Ti, and C and a dual phase at the grain interfaces. Further, we qualitatively estimated the concentration of Ti at the grain boundaries of samples with MXene loadings of 0.5% to 5 wt.% using EDS line scans in SEM. The results showed an increase in normalized Ti intensities with higher MXene concentrations, especially at greater distances from the grain interface, confirming titanium presence with intensified signals (Figure S12, Supporting Information). The TEM micrographs show wrinkling at the grain edges (Figure 4e). This morphology, enlarged in Figure 4f, shows remnants of the layered MXenes morphology upon sintering.

During the process of atomic diffusion, the diffusion of atoms may result in the imprinting of one phase, in this case, MXene, onto ZrB_2_ due to plastic deformation and identical crystal orientations (both MXene and ZrB_2_ exhibit hexagonal crystal structures). Further, the surface energies of TiC and ZrB_2_ are quite close, with values of 2.20 and 2.26 J/m^2^, respectively. This similarity in surface energies minimizes the thermodynamic driving force for the separation of the materials and favors their intermixing.^[^ ^84^, ^85^ ^]^ Observations of similar behavior in non‐UHTCs, such as perovskite materials have shown interdiffusion of the constituent species leading to the formation of wrinkles at grain interfaces.^[^ ^86^ ^]^

**Figure 5:**
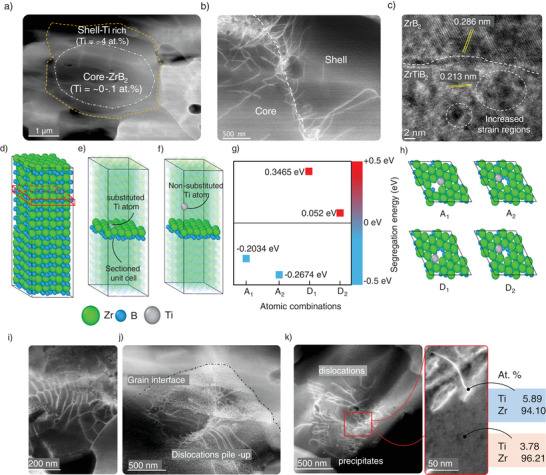
TEM micrographs of ZrB_2_‐1.5 wt% MXene samples showing a) the ZrB_2_ – (Zr,Ti)B_2_ core–shell structure. The shell is highlighted by dislocation tangles and walls at the interface and contains a nominal ≈ 4–5 at.% Ti compared to shell (≈0‐1 at.% Ti). b) TEM micrography of the ZrB_2_ – (Zr,Ti)B_2_ interface at higher magnification. c) high resolution TEM micrograph of the grain boundary interface between two adjacent shells, featured by clean grain boundary and highly strained areas which may be attributed to local Ti enrichment, d‐f) DFT simulation of a ZrB_2_ crystal showing substitution of Ti at preferential sites, g) substitution energy of Ti atom for various cases, h) A_1_, A_2_, D_1_, and D_2_ atomic arrangements. i) polygonized dislocation walls seen in the shell areas of the sintered sample, j) dislocation pile‐up and tangles blocked at the shell interface, k) TEM micrograph showing sporadic precipitation along dislocation arrays seen in the sintered composite with concentration of Ti‐rich precipitates varying between 3.78 to 5.89 at.% as estimated by punctual EDS.

Further, TEM analysis on the ZrB_2_‐MXene (1.5 wt.%) sample confirmed the formation of a (Zr,Ti)B_2_ solid solution shell around pure ZrB_2_ core (**Figure** **5**a), with nominal stoichiometric ratios of Zr between 0.85 and 0.95 in the cationic sublattice and greater dislocation activity at the interface (Figure 5b, Figure S13, Supporting Information). Previous studies have reported such core–shell formation in 3D bulk boride composites, with the principal matrix composition forming the inner core whereas the outer shell is a solid solution decorated with the additive cationic species in the form of precipitates.^[^ ^87^ ^]^

Similar intermixing of Ti and Zr is seen in bulk ZrC‐TiC, ZrB_2_‐TiC, and ZrB_2_‐TiB_2_ systems where an increase in sintering temperatures and dwell times led to the formation of a solid solution phase around the original pristine matrix grain.^[^ ^13^, ^88^, ^89^ ^]^ However, there are no studies that have reported such phase transformation in a 2D additive‐3D boride matrix UHTC. Figure 5c shows the TEM image of a (Zr,Ti)B_2_ solid solution interface with a change in d‐spacings between the two coherent regions (0.286 nm for the core and 0.213 nm for the shell region) along the grain boundary with developed strain regions at the interface (Figure S14, Supporting Information).

We also performed simulation studies to delineate the diffusion propensity of the Ti atoms across the grain boundary of ZrB_2_ and into the bulk structure. Figure 5d shows the unit cell of a ZrB_2_ structure with a section plane along the grain boundary depicted in panels (e and f). By comparing the substitution energies of a Ti atom at the grain boundary (Figure 5e) with those in bulk, away from a grain boundary (Figure 5f) we can understand the energy required for favorable substitutions. This, in turn, helps us to predict the configurations that lead to the formation of cohesive interfaces in these materials. We created a defective grain boundary in the model by generating vacancies along the Zr planes and evaluated the segregation energy of titanium at the grain interfaces. Estimation of the segregation energy at each site indicated that the bulk sites (labeled as D1, D2) exhibited a higher energy barrier (0.475 eV) compared to the grain boundary sites (labeled as A1, A2) on average as shown in Figure 5g,h. Based on these segregation energies, we can conclude that titanium atoms preferentially segregate along the grain boundaries. Further details of the simulation studies are presented in Section S15, Figure S15, and Table S2 (Supporting Information).

The formation of solid solutions must also be explained via thermodynamic assessment by estimating the feasibility of the reaction pathways from reactants to products. We calculated the free energies of formation of the reaction products of ZrB_2_ and TiC as starting materials based on previously published literature.^[^ ^90^ ^]^ The estimated enthalpies and Gibbs‐free energies of the complete displacement reaction between ZrB_2_ and TiC to form corresponding ZrC and TiB_2_ show that the reaction is not feasible at the sintering temperatures (1900 °C) with an estimated Gibbs free energy (ΔG) = +21.9 kJ mol^−1^ (Figure S16a, Supporting Information). However, when the reaction pathways are modified considering the Debye‐Hückel relation to calculate activity coefficients for ideal solutions in solute‐solvent phases (Figure S16b, Supporting Information), the thermodynamic feasibility increases with ΔG < −20 kJ mol^−1^ indicating a conducive reaction pathway toward forming solid solutions of (Zr_1‐x_Ti_x_)B_2_ (Figure S16c, Supporting Information).^[^ ^90^ ^]^

In the TEM micrographs, along the ZrB_2_ core and the solid solution shell, we also observed the presence of dislocations and formation of polygonized dislocation regions at the interfaces. (Figure 5i,j, Figure S17a–c, Supporting Information). This intense dislocation activity is also observed in the SEM using electron channeling contrast imaging (Figure S18, Supporting Information).^[^ ^91^ ^]^ However, TEM allows for images of dislocations to be obtained over a wider range of positive deviations from the Bragg condition.^[^ ^92^ ^]^ Unlike TEM, in SEM the image contrast decreases rapidly with both positive and negative deviations from the perfect Bragg condition,^[^ ^93^, ^94^ ^]^ as opposed to TEM where the depth of focus for objects such as dislocations is very large compared to the thickness of the sample.

Previous studies on bulk ZrB_2_ (3D grains) sintered in the presence of TaSi_2_, MoSi_2_, CrB_2_ or (W/Hf/V)C (i.e., Zr‐Ta‐B, Zr‐Mo‐B, Zr‐Cr‐B, Zr‐W‐B, Zr‐Hf‐B, Zr‐Nb‐B and Zr‐V‐B systems) have reported similar dislocation activity due to the diffusion behavior of the metal species during sintering.^[^ ^87^ ^]^ Dislocation activities in solid solution phases are also reported to lead to strengthening in ZrB_2_,^[^ ^91^ ^]^ especially at elevated temperatures.^[^ ^13^ ^]^ We believe that while Ti atoms preferentially segregate at grain boundaries, their localized accumulation at the grain periphery creates strong compositional gradients. These gradients induce significant local stresses, particularly near the core–shell interface with the core region, composed of pure ZrB₂, directly experiencing these stresses due to differences in atomic radii, expansion mismatches, and chemical potentials between the Ti‐rich boundary regions and the pure ZrB₂ grains.^[^ ^95^, ^96^ ^]^ This stress concentration likely facilitates dislocation nucleation and multiplication within the shell, under specific conditions particularly at high temperatures, potentially enhancing plastic deformation mechanisms.

Small inclusions and precipitates were also observed in the TEM micrographs along the grains within the core–shell interface (Figure 5k) with Ti concentrations varying across the zones ranging from ≈3.5 to 5 at.%. Further, we also observed coffee‐bean contrasts (Figure S17d, Supporting Information) in the shell region, which may be due to these coherent precipitates.^[^ ^97^ ^]^ These 3D islands are also usually associated with stacking faults on (111) planes, which are inclined against each other in the grains.^[^ ^98^, ^99^ ^]^ To our understanding, such contrasting features with a perfect inner crystal structure and stacking faults at the edges are bound by Shockley partial dislocations and are preferably generated along the planes around the vicinity of the additive‐rich regions (in this case: MXene along the grain boundaries).^[^ ^100^ ^]^ To the best of our knowledge, such features have not been observed earlier in 2D‐3D conformal ceramic structures. This observation provides us with a potential for greater control on grain refinement with MXenes as conformal 2D additive materials and consequently aiding toward an increase in the overall strength and hardness of UHTCs through grain engineering.

### 2.5 Mechanical Property Characterizations

**Figure 6:**
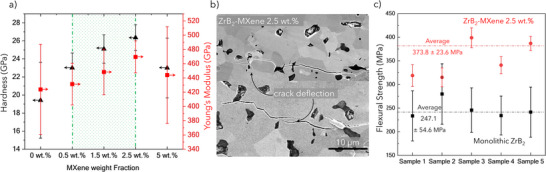
a) Hardness, Young's modulus versus variable MXene weight fraction in sintered ZrB_2_‐MXene composites, b) SEM composite micrograph showing crack deflection and branching (below) in a MXene 2.5 wt.% sample, c) flexural strength of pure ZrB_2_ and ZrB_2_‐MXene (2.5 wt.%) samples.

After demonstrating that the addition of MXenes enhances the densification of sintered samples, reduces oxygen content, and facilitates the formation of a core–shell structure, we investigated the mechanical properties of these UHTCs. Specifically, we assessed the Young's modulus, hardness, indentation toughness, and flexural strengths across varying MXene weight loadings. Hardness measurements, conducted using Vickers microhardness testing and nanoindentation (Table S3, Supporting Information), show a significant improvement from 19 ± 7 GPa for pure ZrB₂ to 26 ± 2 GPa with the addition of 2.5 wt.% MXene (**Figure** **6**a).

The Young's modulus measured via nanoindentation showed a similar trend, with an average modulus of 424 ± 63 GPa for pure ZrB_2_, which increased to 470 ± 22 GPa in ZrB_2_‐2.5 wt.% MXene (Figure 6a, Figure S18a,b, Supporting Information). A notable decrease in the standard deviation was also observed with the addition of MXene, which may be attributed to the improved and homogenized densification of the samples containing MXene. However, a reversal of trends in both hardness and modulus was observed for samples with or more than 5 wt.% MXene, showing 22 ± 5 GPa for hardness and 440 ± 70 GPa for modulus for the 5 wt.% MXene samples. This reversal may be attributed to the evolution of tertiary phases (oxides) that weaken the interfaces.

We also evaluated the crack propagation mode of the Vickers indented samples and observed crack deflection and branching around the grain boundary (Figure 6b). Compared to pure ZrB₂, which exhibited no crack deflection (Figure S19, Supporting Information), the addition of 0.5 wt.% MXene introduced noticeable deviations in the crack path. The presence of localized crack tip blunting and network bridges suggests solid solution strengthening, facilitating efficient energy absorption. Such behavior in crack propagation was observed in (Zr,W)C solid solution systems,^[^ ^101^ ^]^ however, there is no record of such bridging or deflection in 2D‐3D transformed UHTCS to the best of our knowledge. As the MXene content increased from 0.5 to 1.5 wt.% and 2.5 wt.%, more pronounced crack deflections and bridging were observed (Figure S19, Supporting Information). The observed crack deflection and bridging suggest the addition of MXene could lead to improving the fracture toughness of the UHTC and becoming more pronounced at higher concentrations. Further studies are needed to understand the effect of MXene composition, concentration, and sintering temperature on the fracture toughness of ZrB_2_ and other UHTCs.

We, however, note that the magnitude of fracture toughness estimated from secondary crack propagation lengths in indentation tests is low compared to ceramic standards. We have recorded toughness values as high as 7.7 MPa.m^1/2^, over two times of pure ZrB_2_ (with ≈11–15% porosity) in samples containing MXene up to 2.5 wt.% measured across 5 indents and 15 cracks for pure ZrB_2_ and MXene‐ZrB_2_ containing samples. However, measuring secondary crack lengths to estimate the toughness remains challenging especially for new emerging materials since its reliability can be influenced by factors such as accurate crack length measurement, material homogeneity, the indentation rate control, and actual crack propagation within the material. The method relies on empirical equations to relate crack length to fracture toughness, which may not be universally applicable to all materials.

Further, the flexural strength of the samples measured using a three‐point bending method shows a nominal increase in the strength, 247 ± 54 MPa for pure ZrB_2_ to 336 ± 47 MPa for ZrB_2_‐1.5 wt.% MXene (Figure 6c) and 373.8 ± 23.6 MPa for ZrB_2_‐2.5 wt.% MXene. Table S3 (Supporting Information) summarizes the mechanical properties of the sintered samples measured at room temperature conditions. Table S4 (Supporting Information) further shows the variance in the flexural strengths of the UHTC measured across 5 samples using 3‐point bending tests. We believe that further characterizations are needed to assess the overall effect of MXene on the flexural strength using the 4‐point bending method, to evaluate the crack deflection behavior specifically to understand the role of solid solutioning toward crack propagation, and the fracture toughness of these novel composite structures.

Our assessment of the measured properties suggests that the improvement in the mechanical behavior of the ZrB_2_‐MXene sintered samples is due to MXene's ability to affect the sinterability of the green bodies, resulting in denser samples. However, the core–shell and solid solution formations with the presence of dislocations with phase‐transformed MXenes‐ZrB_2_ interfaces acting as dislocation sinks, indicate that the reactions and phenomena caused by nanometer‐thin MXene flakes at the grain boundaries may affect the properties of the resulting UHTCs. The presence of dislocation arrays in sintered ZrB_2_‐MXene (and ceramics, in general) may aid toughening by enabling some degree of plastic deformation, particularly at high temperatures.^[^ ^102^ ^]^

Design and engineering of microstructures with controlled core–shells and dislocations in ceramics with MXenes as the additive nanomaterials can improve strength and toughness, thus widening the application range of MXene‐derived UHTCs. This is the first step toward understanding the microstructural evolution and the structure‐property relationship in these new MXene‐derived UHTC structures.

## 3 Conclusion

This study evaluated the potential of MXenes as additives in developing advanced ultra‐high temperature ceramics (UHTCs). Leveraging MXenes offers a distinct advantage in nanoscale structural design due to their compositional similarity to bulk UHTCs, nanometer thicknesses, hydrophilic surfaces, and colloidal stability without any surfactants. We further demonstrated the adaptability of Ti_3_C_2_T*_x_* MXenes, particularly with ZrB_2_ as the principal matrix, through a scalable electrostatic self‐assembly process. This approach yields homogeneous green bodies without the need for surfactants, utilizing the inherent surface charges of MXenes.

The synthesis and sintering processes used in this study resulted in enhanced density, reduced oxygen content in the ZrB_2_ matrix, and overall improved mechanical properties of the UHTCs. MXene addition also exhibited intriguing microstructural features, notably the formation of a distinctive core–shell matrix, with greater dislocation activity observed along the core–shell interface, and the shell consisting of (Zr,Ti)B_2_ solid solution. The observed improvements in hardness, Young's modulus, and flexural strength in the presence of MXene highlight its multifaceted role as a sintering aid, reducing agent, and potential reinforcing material. These findings are translatable to other MXene‐UHTC systems, further allowing us to a tailored design of UHTCs with MXenes as reinforcing materials.

## 4 Experimental Section

Nanosized ZrB_2_ (750 nm) was procured from US‐Nano, TiC (325 mesh), Ti (325 mesh), Al (325 mesh), HCl (12 M), HF (48 wt.%), LiCl (99.9%), were procured from Fisher Scientific. ZrB_2_ powders were stored in a glovebox to avoid contamination. All reagents and materials were used as obtained unless specified in the experimental protocols.

### Synthesis of MAX Phase Precursor (Ti_3_AlC_2_)

The MAX phase precursor was synthesized via reactive sintering of the elemental powders of TiC:Al:Ti mixed in a ratio of 2:1:1. For example, to synthesize a MAX Ti_3_AlC_2_ of mass 50 g, 30.77 g TiC, 6.93 g Al, and 12.3 g Ti powders were mixed. The protocols for synthesis are identical to the previously reported methods.^[^ ^52^ ^]^ In brief, elemental powders were mixed in a tumbler mill with zirconia balls for 10–12 h. There was no effective size reduction of the particles during ball milling. The mixed powders were loaded in an alumina crucible, covered with a graphite foil lid, and sintered in a tube furnace at atmospheric pressure, in an inert gas (argon) environment. The optimized sintering temperature was 1400 °C with a dwell time of 4h. The heating rates were 3.5 °C min^−1^, and the cooling rates were 5 °C min^−1^. Upon synthesis, the MAX phase was milled into fine powders and sieved with a 70 µm sieve (the average particle sizes were 20–70 µm) for the subsequent steps of etching and delamination.

### Synthesis of MXene (Ti_3_C_2_T_x_)

For synthesizing MXene, in a typical experiment, 1 g of the MAX phase was etched in a 30 mL etching solution (18 mL 12 M HCl + 3 mL 48% (wt./wt.) HF + 9 mL DI water). This mild etching method uses 10% HF by concentration for MXene synthesis with HCl:HF:H_2_O in a ratio of 6:3:1 by volume. The etching was carried out in a fume hood on a stir plate at 35 °C and 300–400 RPM speeds, for 24 h in an oil bath.

Etched powders were then washed to neutrality with de‐ionized water in a centrifuge at 5000 RPM (≈3500 RCF). In a rough estimate, 200–250 mL water was used to bring the pH to 6–7. These washed powders were then delaminated with 1 g LiCl (0.0237 mol) in 50 mL water (for 1 g of etched MXene) for 4 h at 65 °C with continuous stirring in an oil bath, with continuous argon flow in the container. Post LiCl treatment, the delaminated MXene was transferred to centrifuge tubes and washed at 14 000 RPM (≈23 000 RCF) for 5 min. The cycle was repeated three times to remove excess intercalants.

After this step, the delaminated MXene clay was diluted with 30–40 mL of water and allowed to sit on the lab bench in a dark place overnight (12–16 h). It was observed that allowing the MXene to bloom resulted in highly concentrated MXenes with considerably greater yields. Separation of the single flakes MXene was carried out by carefully prodding the overnight bloomed clay with a glass rod and shaking it in an automatic vortex shaker. The solution was then centrifuged at 2380 RCF for 30 min, which yielded single flake MXene of concentrations between 5 and 30 mg mL^−1^ (depending on the amount of dilution). The overall yield of the MXene with this method was ≈83%.

### Zeta Potential Measurements

The zeta potentials of ceramic suspensions and Ti_3_C_2_T*_x_* MXene were measured with a Malvern Zetasizer Nano ZS instrument equipped with a 632 nm HeNe laser at a detector angle of 173° using a disposable folded capillary cell. ζ potentials were estimated by mapping the electrophoretic mobility using the Helmholz‐Smoluchowski (HS) model for electrophoresis with proprietary Malvern software.^[^ ^103^ ^]^ The suspensions were stabilized for 30 s before measurement in ambient conditions. Three sets of 20 runs each were taken and averaged to obtain the zeta‐potentials. The concentration of the MXene and ceramic suspensions was 0.01 mg mL^−1^.

### Preparation of Ceramic Green Bodies (Ti_3_C_2_T_x_ + ZrB_2_ Mixtures)

The protocol consisted of three main parts: the first part of the protocol involved preparing the ZrB_2_ suspension. ZrB_2_ powders (USNano‐750 nm size) were measured by mass and added to a small glass vial. DI water was added to the vial at a ratio of 5 mL for every 1 g of ZrB_2_ (concentration: 200 mg mL^−1^). The mixture was then mixed well, and probe/bath sonicated for 30 min. Using a 0.1 M HCl solution, the pH of the ZrB_2_ suspension was lowered to pH 5.

In the second part of the protocol, Ti_3_C_2_T*_x_* MXene was measured according to the calculated amount (*x* wt.% of ZrB_2_) where x = 0.5 to 15 wt.% and was added to a 50 mL centrifuge tube. The tube was then probe sonicated for 15 min with a 1/2′’ tip at 40% amplitude (60 µm) and 500‐watt power, in an ice bath. After sonication, the MXene was transferred to a glass beaker/flask and diluted with 0.5 mL DI water for every 1 mg of MXene. The pH of the MXene suspension was then lowered to pH5 with a 0.1 M HCl solution.

In the third part of the protocol, the ZrB_2_ slurries and MXene suspensions were mixed. The ZrB_2_ slurries were added dropwise to a continuously stirring MXene suspension at a rate of ≈100 mg min^−1^. The stirring speeds were optimized at 700 RPM and the mixture was stirred for 1 h at room temperature. Post stirring, the suspension was rested for 10–15 min for the phases to separate. The solvent was pipetted out and the green bodies were vacuum filtered with an 800 nm MCE (mixed cellulose ester) membrane. The powders were then dried at 200 °C for 12–18 h in continuous vacuum.

While higher stirring speeds do decrease separation time, they introduce significant processing challenges that can compromise safety, equipment integrity, and mixing effectiveness. Lower speeds, on the other hand, extend processing times and may not provide sufficient mixing though equally effective. Therefore, a stirring speed of 700 RPM was chosen as the optimal compromise, ensuring efficient mixing while minimizing processing difficulties.

This selection was based on a comprehensive evaluation of the mixing processes, considering both the physical limitations of the equipment and the practical aspects of laboratory operation. By choosing 700 RPM, it was ensured that the mixing process was efficient, reproducible, and safe, leading to reliable and consistent results in the experiments.

### Estimating the Volume Fraction of MXene in ZrB_2_

The weight loadings of MXene in ZrB_2_ were estimated using the rule of mixtures.

The density of MXenes (4.22 g cc^−1^) and ZrB_2_ (6.08 g cc^−1^) were used to estimate the volume fraction and the weight fractions of the MXene for a 10 g green body mass. For example, to make a 1.5 wt.% MXene in ZrB_2_ green body of total mass 10 g, 150 mg of MXene was needed.

For a given MXene solution of concentration 10 mg mL^−1^, 15 mL of MXene solution was needed in a ZrB_2_ powder slurry of mass 9.85g.

Note: The di‐boride powders often come with oxide impurities. The oxides may be mitigated via:

1. washing in methanol
2. introducing dwell steps during sintering to remove volatile boria (B_2_O_3_) in vapor forms.

### Sintering of ZrB_2_‐MXene Ceramics by Field Assisted Sintering (or Spark Plasma Sintering (SPS))

The dried green bodies were sintered in a 20 mm graphite die lined with graphite foil. The punches were protected with boron nitride coated graphite foil (140 µm thick). Sintering was carried out in a helium atmosphere. The chamber was set to a vacuum (2–3 Pa) and held for 15 minutes before inert gas backfilling. 1.5 g of the green bodies were loaded in a die and ramped to 1200 °C at 70 °C min^−1^ with 30 MPa pressure applied at room temperature. The powders were soaked at 1200 °C for 1h. Further, the pressure increased to 50 MPa at 10 MPa min^−1^ and 1900 °C at 70 °C min^−1^ and dwell for 15 min. The sintering regimes are provided in Figure S6 (Supporting Information).

### Characterization and Sample Preparation

**Densities** and porosities were measured via Archimedes’ method upon immersion in distilled water. **Phase composition** was determined via 2D and 1D XRD methods with a Bruker Discover D8 diffractometer, in a range of 2°–70° 2θ, for MXene and MAX phase, and 2°–90° 2θ, for the sintered composites. The step size for the 1D method was 0.01° with 1.2 sec step^−1^. **Polishing and grinding** of the samples mounted in epoxy were carried out with silicon carbide paper (180, 320, 600, 800, and 1200 grit sizes) until a mirror finish was obtained. **The microstructure** of the ceramics, MXene, and the MAX phases was examined with SEM (5 kV and spot size of 6 for secondary electron imaging), (5 kV and spot size of 9–11 for BSE imaging).

Further, the areal porosities were estimated via the back‐scattered electron imaging (BSE‐SEM) method followed by an image segmentation technique to identify average pore areas (Figure S20, Supporting Information). Associated Table S5 (Supporting Information) shows the total pixels of the entire region (green + red) with the red zones indicative of the pores which are subdivided based on the average darkness of the field from the BSE micrographs. The average pore area (represented as null area%) decreases from 14% to 11% in samples with 0.5 wt.% MXene. A further decrement is observed in samples with 1.5 wt.% (6%) and 2.5 wt.% (7%) while the samples with 15 wt.% MXene exhibited a trend reversal with the average null areas increasing to 14%.

Further TEM studies were performed on selected samples. A TEM specimen was prepared by cutting a 3 mm wide disc from the bulk material and mechanically grinding it down to a thickness of about 100 µm. Then, an 80 µm deep bowl was milled in the center of the disc using a dimpler equipped with a diamond covered wheel leaving a thickness of about 20 µm in the center. The final thinning was achieved using a Precision Ion Polishing System (PIPS) at 5 KeV with an argon beam until the incipient perforation occurred. Local phase analysis was carried out using a Transmission Electron Microscope (FEI Tecnai F20 ST TEM) with a nominal accelerating voltage of 200 kV and equipped with energy dispersive X‐ray system (EDS, TEM Image Analysis (TIA) version 3.2 sp6) with and ultra‐thin window. Electron diffractions were resolved through the commercial software JEMS (Java Electron Microscopy Software, P. Stadelmann, Switzerland).

**Mechanical property** measurements (microhardness and indentation toughness) were performed with a Vickers microhardness indenter with a load of 9.8N and a dwell time of 15s. Eight indentations at a spacing of 2.5d (d = the length of the indent diagonal) were taken from each sample and the hardness was averaged. The fracture toughness of the samples was measured by estimating the crack length propagated from the indention generated from hardness measurements. The Anstis equation was employed for calculating the fracture toughness:

(1) $$K_{\mathrm{lc}}=0.016\times {\left[\frac{E}{H}\right]}^{0.5}\times \left(\frac{P}{c^{1.5}}\right)$$

Flexural strength of the samples was measured using a 3‐point bending method on polished cuboidal samples of dimensions 10 mm × 5 mm × 1 mm.

**Nanoindentation tests** were conducted using a Hysitron TI 980 (Hysitron, Inc., Minneapolis, Minnesota) nano‐indenter equipped with a three‐sided pyramidal Berkovich diamond indenter tip. The sample was cold mounted in epoxy and polished to 2400 grit followed by the specimen being washed in water bath to remove any dust on the sample. The indentation tests were carried out in load‐controlled mode with a constant strain rate of 0.5 S^−1^ and a maximum load of 10 mN. The 5 × 5 grid of indents was carried out on the sample with indentation spacing at least 10 times the indentation depth to avoid the influence of elastic‐plastic zone from adjacent indents. The stiffness of the sample from the indentation test was analyzed using the system's inbuilt quasi‐static analysis package, which extracts reduced modulus and hardness of the sample from the force versus displacement curve. Further, the elastic modulus was calculated using the formula:

(2) $$\frac{1}{E^{*}}=\frac{1-\upsilon^{2}}{E}+\frac{1-{\upsilon_{i}}^{2}}{E_{i}}$$

where E* is the reduced modulus obtained from indentation data, E is the reported elastic modulus of the specimen, E_i_ = modulus of the Berkovich indenter, ν and ν_i_ are Poisson's ratio of the specimen and the indenter, respectively. The Young's modulus and Berkovich indenter Poisson's ratio was 1147 GPa and 0.07, respectively. Young's modulus was estimated by calculating the contact stiffness(S) as the slope of the unloading curve (S = dP/dh) at the maximum depth h_max_ in the load–displacement curve. The Poisson's ratios of the samples were estimated using the rule of mixtures, with ν_MXene_ = 0.241 and ν_ZrB2_ = 0.11.^[^ ^104^, ^105^ ^]^

All mechanical properties were measured on samples derived from the same master batch of green bodies, ensuring consistency in material processing and eliminating variations due to differences in sample preparation and to maintain experimental accuracy. For example, in a typical batch processing, 6g  of green bodies were synthesized to be sintered further into 4 samples for each MXene wt.% loading.

**DFT Simulations** Density functional theory (DFT) calculations were completed in the Vienna ab initio simulation package (VASP),^[^ ^106^ ^]^ using the projector augmented wave method (PAW)^[^ ^107^ ^]^ and the Perdew, Burke, and Ernzerhof (PBE) exchange correlation functional.^[^ ^108^ ^]^ The plane wave basis set was selected with a 520‐eV cutoff. Using the conjugate gradient method, geometry relaxation was executed with a 0.002 eV/Angstrom force threshold on the ions and a 10e6 eV threshold energy for electronic self‐consistent convergence. Due to the large supercell in real space, the Brillouin Zone was sampled with a single k point centered at gamma. The utilized cell dimensions were 16.78 × 16.78 × 50.52 angstroms. The grain boundary structures were created using the integrated GUI created by Zheng et al.^[^ ^109^ ^]^ This created a sigma 7 grain boundary (GB) unit cell with a 21.79‐degree twist and coherent periodic boundary conditions. More details are provided in Section S15 (Supporting Information).

## Conflict of Interest

The authors declare no conflict of interest.

## Author Contributions

S.K.N. and B.A. planned and visualized the study. S.K.N. designed the experiments. S.K.N., Y.I., A.V., and N.C.B.S. synthesized the precursor MAX phases and the MXenes, Y.I. prepared and optimized the ceramic green body synthesis protocols under the mentorship of S.K.N. Y.I., and A.V. assisted S.K.N. in sample preparation for characterization. B.C.W. assisted with the X‐ray diffraction data. N.G. and L.S. performed the transmission electron microscopy (TEM) characterizations. S.K.N., N.G., and L.S. analyzed the TEM data. S.G. performed the density functional theory studies under the supervision of G.T. and assisted in writing and visualizing the simulation data. A.K. conducted the nanoindentation tests under the supervision of N.C., S.K.N., and A.K. analyzed the nanoindentation data. The manuscript was drafted by S.K.N. and revised by B.A. with revisions and input from all authors.

## Supporting information



Supporting Information

Supplemental Video 1

## Data Availability

The data that support the findings of this study are available in the supplementary material of this article.
