# Supplementary material for: Ti3C2T x MXene‐Zirconium Diboride Based Ultra‐High Temperature Ceramics
Source: Adv Sci (Weinh). 2025 Apr 30;12(23):2500487. doi: 10.1002/advs.202500487 (PMC12199373; doi:10.1002/advs.202500487)
Supplement: Supplementary file 1 — Supporting Information [file ADVS-12-2500487-s002.docx]

Supplementary Information

**Ti_3_C_2_T*_x_* MXene-Zirconium Diboride Based Ultra-High Temperature Ceramics**

*Srinivasa Kartik Nemani^1^, Nicola Gilli^2^, Steven Goldy^3^, Ankit Kumar^4^, Yooran Im^3^, Austin J. Vorhees^1^, Brian C. Wyatt^4^, Nithin Chandran^1^, Nikhilesh Chawla^4^, Garritt J. Tucker^5^, Laura Silvestroni^6^, Babak Anasori^1,4^,**

1. *School of Mechanical Engineering, Purdue University, West Lafayette, IN, 47907, USA.*
2. *CNR-ISMN Institute for Nanostructured Materials, Via Gobetti 101, Bologna, 40129, Italy*
3. *Colorado School of Mines, Golden, CO, 80401, USA.*
4. *School of Materials Engineering, Purdue University, West Lafayette, IN, 47907, USA.*
5. *Department of Physics, Baylor University, Waco, TX, 76706, USA.*
6. *CNR-ISSMC Institute of Science, Technology and Sustainability for Ceramics, Via Granarolo 64, Faenza, 48018,  Italy*

** Email: banasori@Purdue.edu*

**Figure S1**


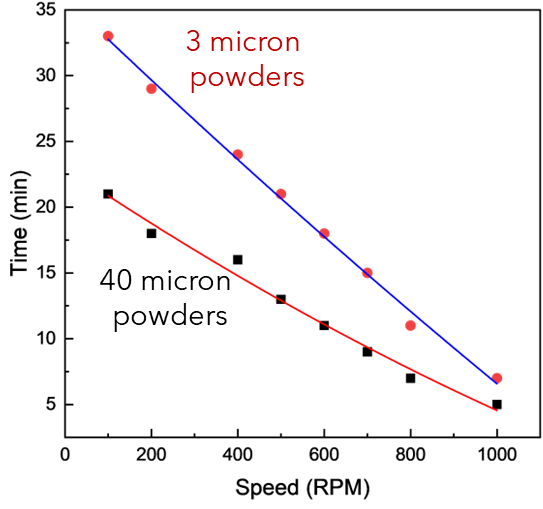


**Figure S1**: a) Settling time vs mixing speed (RPM) for the green bodies.

**Figure S2**

**
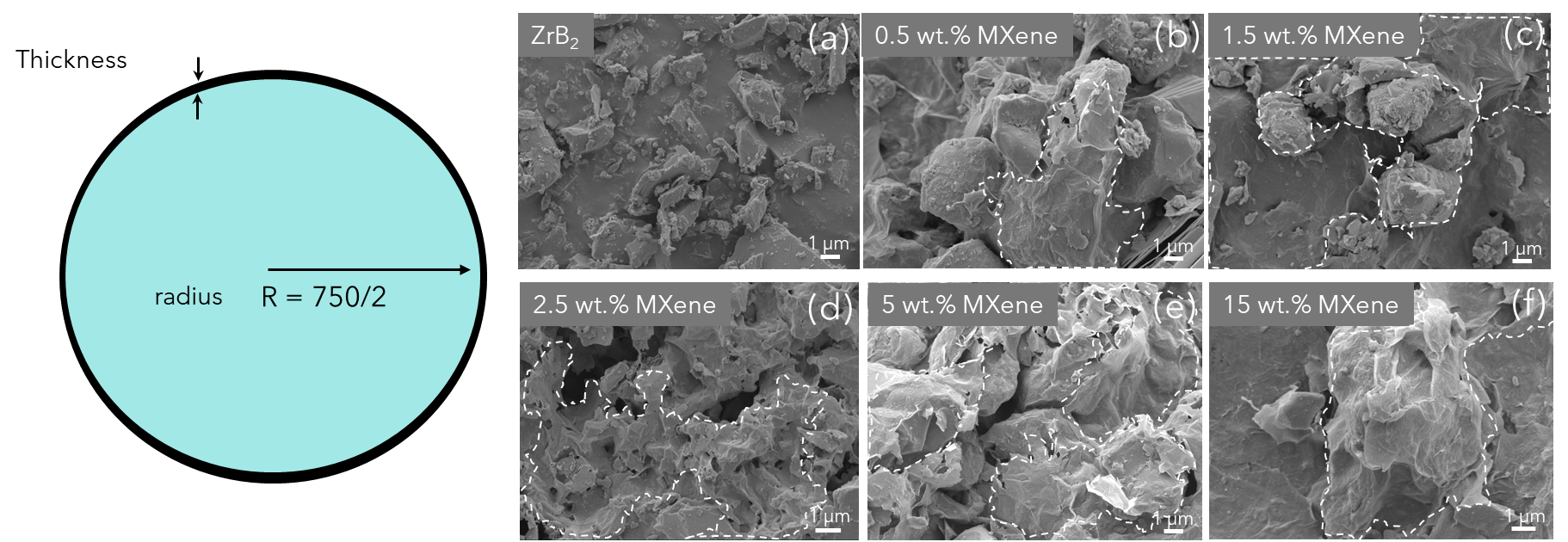
**

**Figure S2**: Geometrical analogue of a spherical ZrB_2_ grain (cyan color) covered with a MXene sheet (black) a-f: SEM micrographs of ZrB_2_ (a) and ZrB_2_-MXene green bodies with increasing MXene content (b-f).

**Figure S3**


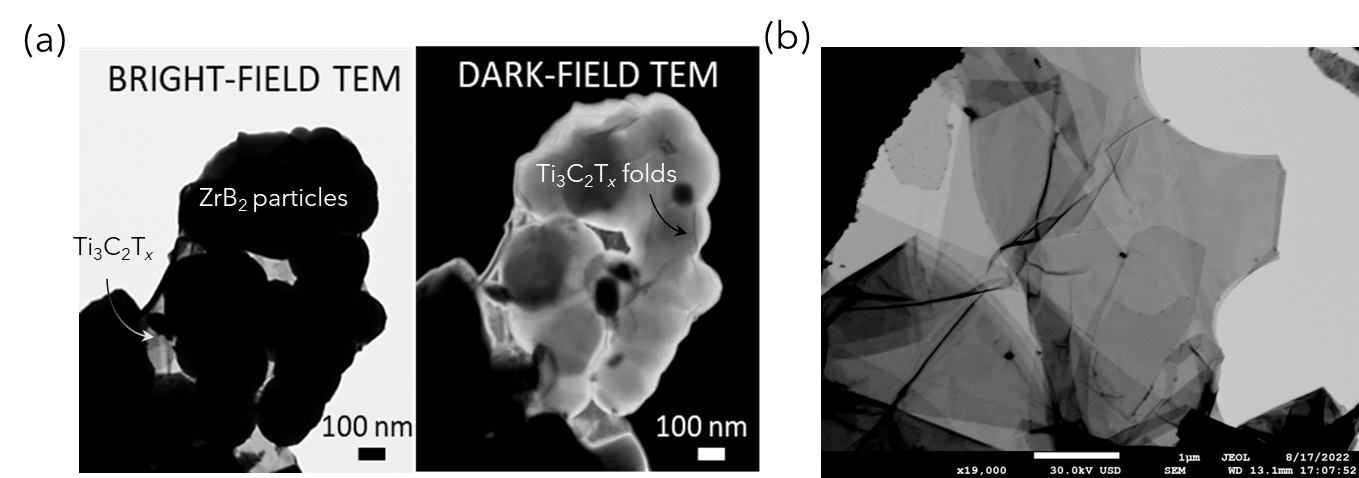


**Figure S3**: a) Bright-field and dark field TEM (30kV) micrographs of a 1.5 wt% MXene-ZrB_2_ green body, b) TEM micrographs of single to few layers delaminated Ti_3_C_2_T*_x_* MXene.

**Figure S4**


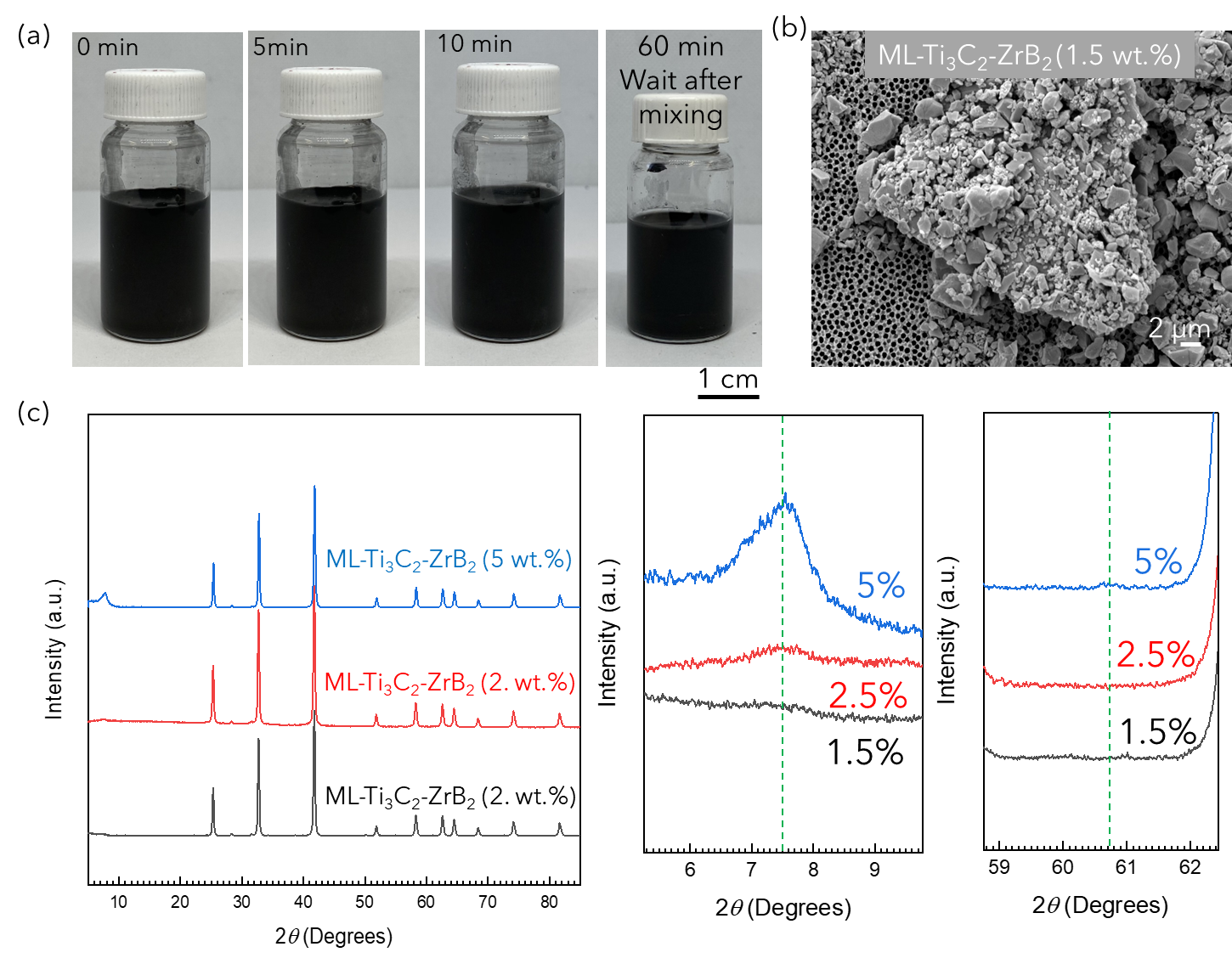


**Figure S4**: a) Multi-layer MXene-ZrB_2_ attempted green bodies via self-assembly. We do not observe any self-assembly of the particles as seen in single-/few-layer MXene-ZrB_2_ system. b) SEM micrograph of the ML-MXene particles and ZrB_2_ grains (No conformal coverage observed). c) XRD patterns for ML MXene-ZrB_2_ green bodies. This indicates that there is an eventual solute-solvent phase separation due to gravity (The density of ZrB_2_ is 6.08 g/cm^3^ and Ti_3_C_2_ MXene is 4.2 g/cm^3^). The XRD patterns show intense peaks for the (002) plane of MXene in the mixed green bodies for 1.5 wt.% multi-layer MXene-ZrB_2_ powders with higher intensities seen for samples with multi-layer 2.5 wt.% and 5 wt.% MXene concentrations.

**Figure S5**


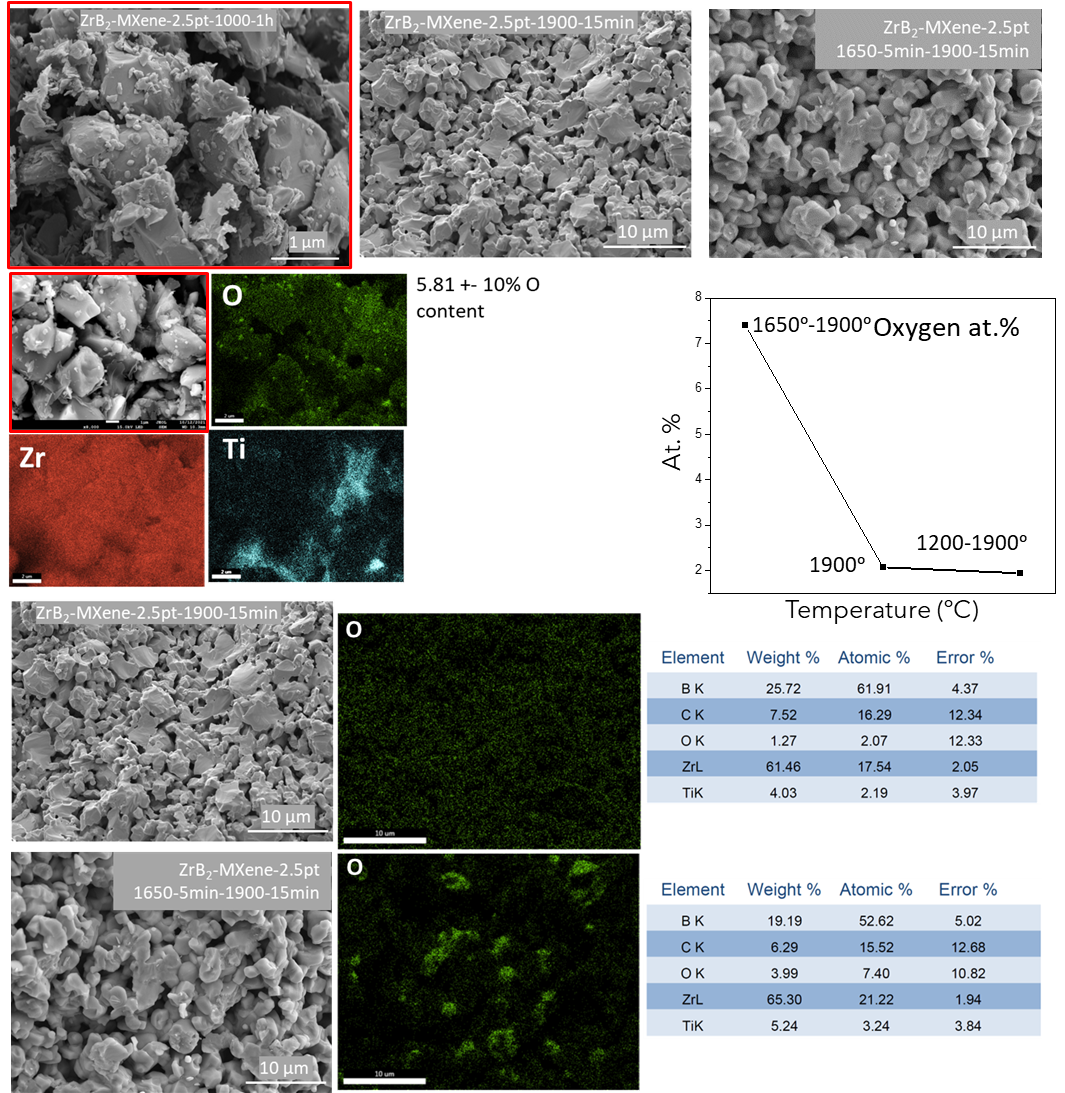


**Figure S5:** SEM micrographs and EDS data of sintered samples with different sintering conditions and sintering times.

**Figure S6**


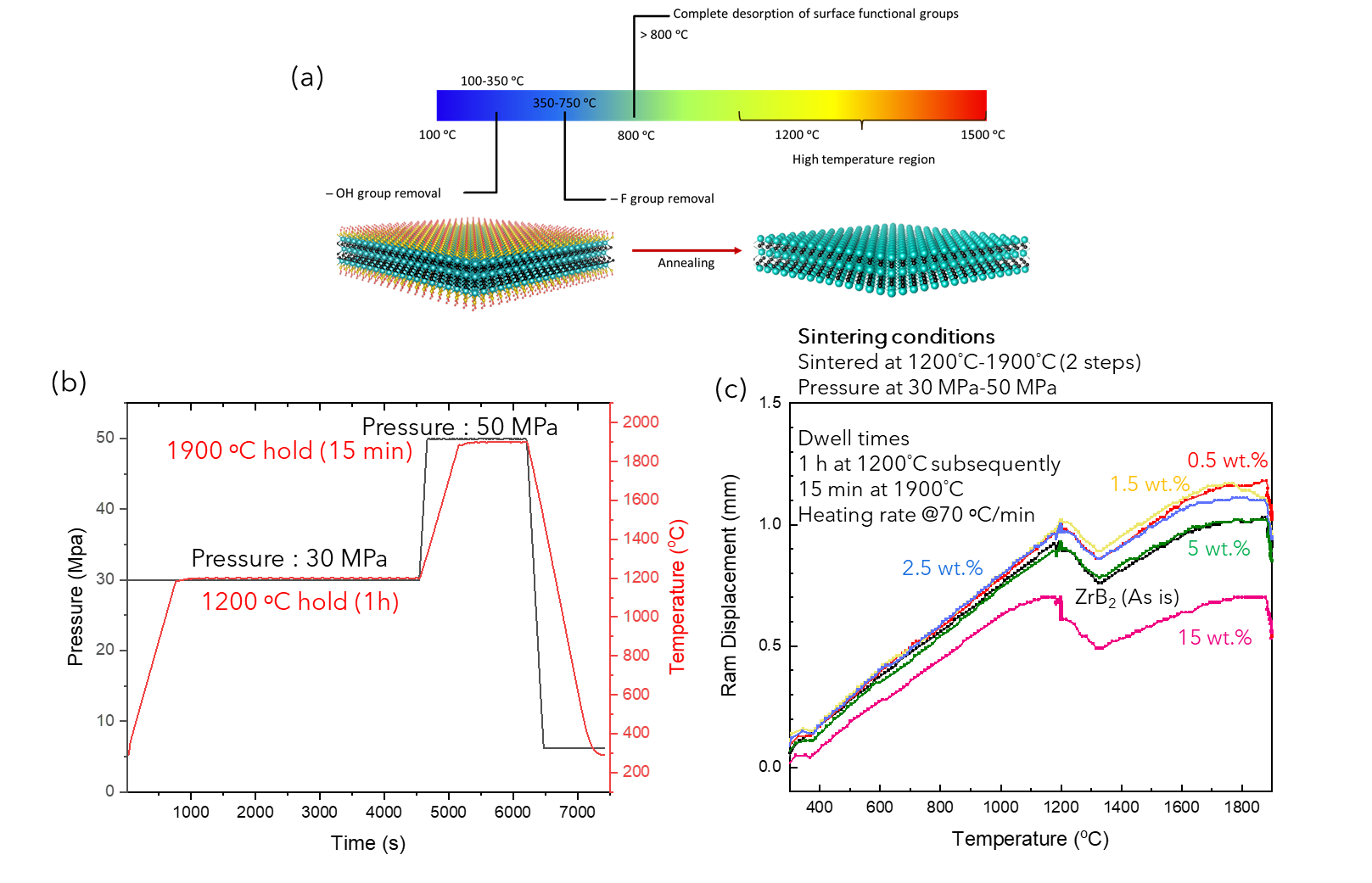


**
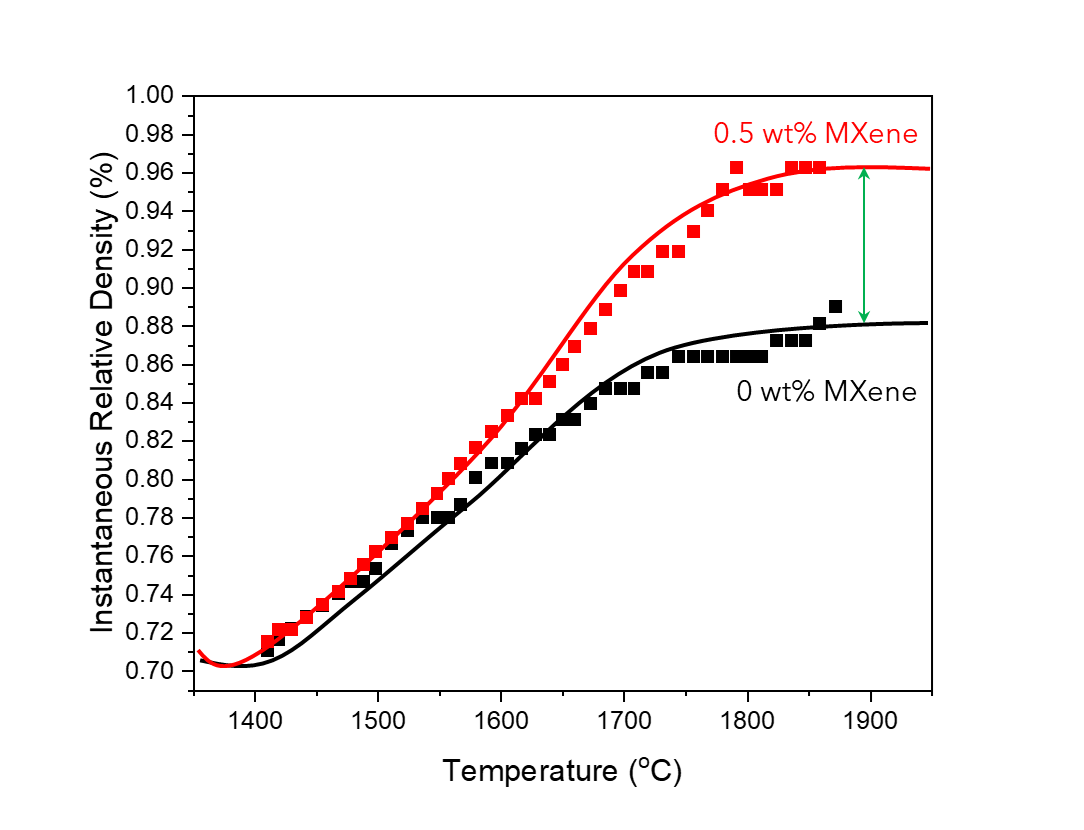
**

(d)

**Figure S6:** a) Schematic diagram showing MXene phase transition as a function of temperature, MXene transforms to a cubic TiC_y_ structure above 1200 ºC, b) Sintering parameters in SPS, c) Ram displacement vs temperature plot for various green bodies. d) Instantaneous density (%) vs Temperature plot for pure ZrB_2_ and with MXene addition.

**Supplementary text** **S6**

The sintering regimes for densification of the green bodies were designed based on our previous report showing complete phase transformation of MXene to TiC*_y_* phase above 1200 °C.^[1]^ Subsequently, a two-step sintering regime was designed for densifying the composites. Green bodies were loaded in a graphite die and a uniaxial pressure of 6 MPa was applied at room temperature and pressure. The chamber was evacuated to 2-3 Pa in 10-20 minutes upon which the chamber was backfilled with high purity helium. The gas flow rates were adjusted to 0.5 lpm. Next, the pressure was increased to 30 MPa upon which the temperature was increased at a rate of 70 °C/min till 1200 °C where the die was soaked for 1h. Post 1200 °C treatment, the pressure was increased to 50 MPa at a rate of 20 MPa/min (2 minute) while the temperature was increased at 70 °C/min to 1900 °C. The pressure was applied before the temperature reached 1400 °C beyond which typical densification regimes for ZrB_2_ are reported. The samples were held at temperature 1900 °C with 50 MPa pressure for 15 min. Upon sintering, the die was cooled at 100 °C/min till 700 °C and then naturally cooled to room temperature. The entire sintering and cooling process was carried out in an inert helium atmosphere.

*Estimation of instantaneous densities*

Figure S5-C shows the ram displacement (in mm) vs temperature (°C). Real time displacement was tracked by observing the initial and final ram displacement points starting from room temperature till sintering temperature (1900 °C).

The instantaneous densities were estimated using the following expression:

$$\rho=\rho_{final}.\left(\frac{h_{o}}{\Delta h}\right)$$

Where $\rho$is the instantaneous density,$\rho_{final}$is the final density, h_0_ is the final thickness and $\Delta h$is the change in ram displacement during sintering. **Figure S6d** shows the instantaneous relative density vs temperature for a ZrB_2_ (as is) and MXene (0.5 wt.%)-ZrB_2_ ceramic sintered via SPS. The Difference in relative densities at 1900 ᵒC post sintering is estimated to be ~6.7% (89% for monolith vs 95-96% for the Ti_3_C_2_-ZrB_2_).

The average densification rate (s^-1^) for pure ZrB_2_ was ~0.00300 s^-1^ ± 0.00027 while an increase was observed for samples with 0.5 wt.% MXene addition. The peak displacement rates were 0.00430 ± 0.00023 s^-1^ for 0.5 wt.%, 0.00450 ± 0.00033 s^-1^ for 1.5 wt.%, 0.00390 ± 0.00031 s^-1^ for 2.5 wt.% and 0.00350 ± 0.00029 s^-1^ for 5 wt.% samples, respectively (**Figure 3b** in the main manuscript).

**Figure S7**


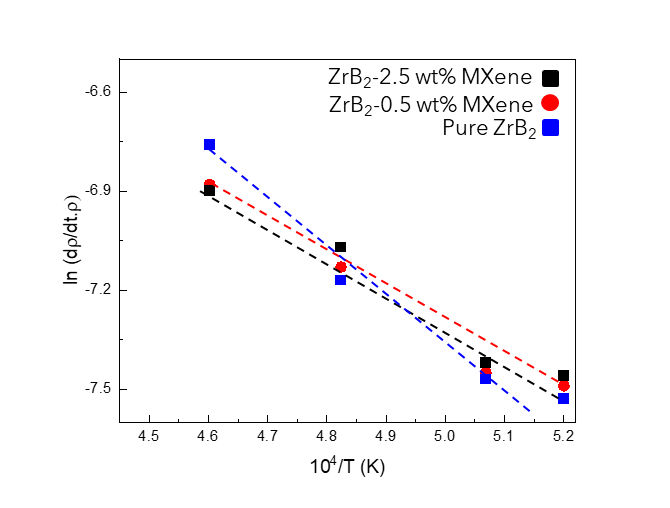


**Figure S7**: Log of instantaneous densities vs Inverse of temperature plot to estimate activation energies.

**Supplementary text S7**

For estimating the activation energies in the sintering process, we adapted Coble and Herring’s model for grain boundary/lattice diffusion which was previously implemented by Lonergan et al. for estimating sintering behavior of ZrB_2_ during reactive hot-pressing.^[2]^

Specifically, for the grain boundary creep in the system:

$$\varepsilon=N\frac{D\delta\sigma\Omega}{G^{3}\kappa T}$$

Where $\varepsilon$ is the strain due to grain boundary diffusion,$N$the diffusion constant,$D$ the diffusion coefficient, $\delta$grain boundary width, $\sigma$applied stress, $\Omega$ unit cell volume, $G$is the grain size, $\kappa$ Boltzmann constant and $T$the sintering temperature.

creep which is equivalent to the linear strain may be equated to densification rates expressed as:

$$\varepsilon'=-\frac{1}{l}\frac{dl}{dt}=\frac{1}{\rho}\frac{d\rho}{dt}$$

Where $\varepsilon'$ is the linear strain rate $l$ is the thickness of the green body compact, and $\rho$ is the density of the sample.

Previous studies have shown that in the ZrB_2_ system, the densification driving forces are primarily surface energy reduction, which is a function of the pore curvature (K) and surface energy ($\gamma$) expressed as $K\gamma$, and accompanied by stresses at the pores $\sigma_{p}$ (along the grain boundaries). The densification rate $\frac{1}{\rho}\frac{d\rho}{dt}$may be then expressed as:

$$\frac{1}{\rho}\frac{d\rho}{dt}=N\frac{D\delta\sigma\Omega}{G^{3}\kappa T}\left(\sigma_{p}+K\gamma\right)$$

Here, we assumed that all the parameters are constant with only variable being temperature (T), which establishes estimation of diffusion coefficient (D) using Arrhenius equation:

$$D=D_{o}\exp\left(-\frac{Q}{RT}\right)$$

Where D*_o_* is the diffusion coefficient constant, Q is the activation energy, and R is the ideal gas constant (kJ/mol). By substituting the above expression in the equation (3), the diffusion term may be represented in the form $y=mx+C$:

$$\frac{1}{\rho}\frac{d\rho}{dt}=B\exp\left(-\frac{Q}{RT}\right)$$

Where B is a term independent of temperature, based on the above assumption. This expression can then be plotted as a function of the densification rate $\frac{1}{\rho}\frac{d\rho}{dt}$ vs inverse of temperature $\frac{1}{T}$wherein the slope may then be equated to$\left(-\frac{Q}{R}\right)$ (**figure S6**). It is seen that the slope in figure S5 is not changing which indicates a single dominant diffusion mechanism (grain boundary diffusion in this study) as a primary driving factor for densification at sintering temperatures of 1900 ᵒC.^[3]^ Diffusion kinetics are largely dependent on the sintering temperatures. A grain boundary dominant diffusion is expected to be translative to other MXene-ceramic matrix systems too, as presence of a sub-stoichiometric phase with a large aspect ratio (2D MXene derived carbide/nitride) at the grain interface promotes diffusion of species due to decrease in associated surface energies and presence of conducive diffusion pathways due to vacancies in the structure.

**Figure S8**


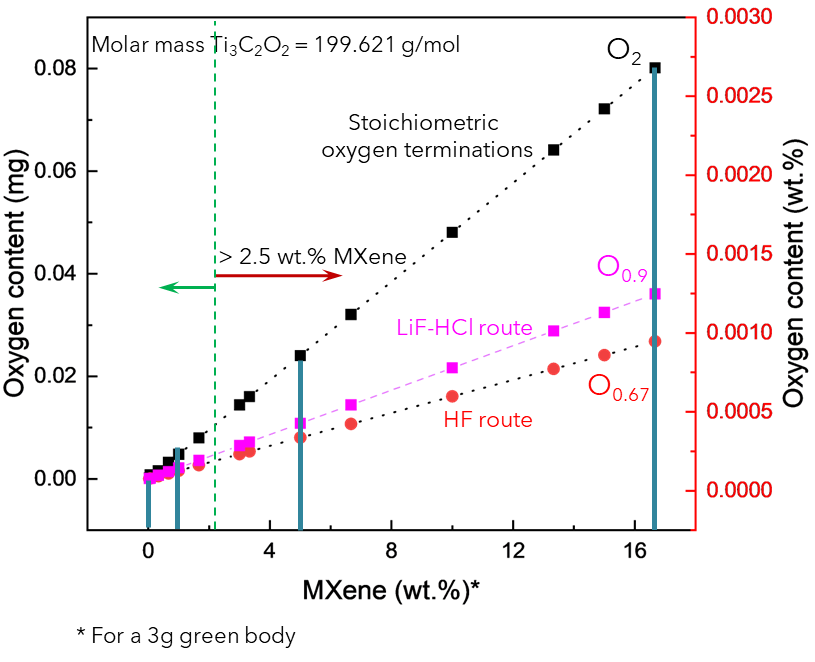


**Figure S8**: Oxygen content vs MXene (wt %) in MXenes synthesized via various synthesis processes. The vertical green (dotted) line represents the threshold for optimum MXene wt.% for ideal property in the UHTC samples.

Net oxygen content (mg) and (wt.%) (as measured by XPS and NMR studies) vs MXene (wt.%) plot for a 3g green body. The net oxygen stoichiometric ratios are obtained from previous NMR and XPS studies quantifying the oxygen terminations in a Ti_3_C_2_T*_x_* MXene. We have assumed that the OH terminations too are analogous to oxygen terminations due to our processing steps where the green bodies are treated at 200 °C in vacuum to remove water entrapped in the powders. The **black dotted line** represents Ti_3_C_2_O_2_ MXene where the entire basal plane is assumed to be oxygen terminated. Further, we have also estimated the net oxygen contribution (from the surface terminations) for three etching processes of the Ti_3_AlC_2_ stoichiometric MAX phase:

LiF-HCl MILD etching route: Ti_3_C_2_**(OH)_0.06_**F_0.25_**O_0.84_** (Total O: 0.9)^[4]^ Pink dotted line

HF synthesis: Ti_3_C_2_**(OH)_0.12_**F_0.8_**O_0.54_** (Total O: 0.66)^[4]^ Red dotted line

HF-HCl method: Ti_3_C_2_**O_0.254_OH_0.456_**F_1.218_Cl_0.072_ (Total O: 0.71)^[5]^

**Note**: The OH and O terminations are estimated as O*_y_* in the oxygen wt.% calculations

The net oxygen contribution in wt.% from the surface terminations is estimated to be >0.05 mg (~0.002 wt.% of the total green body mass).

**Figure S9**

**
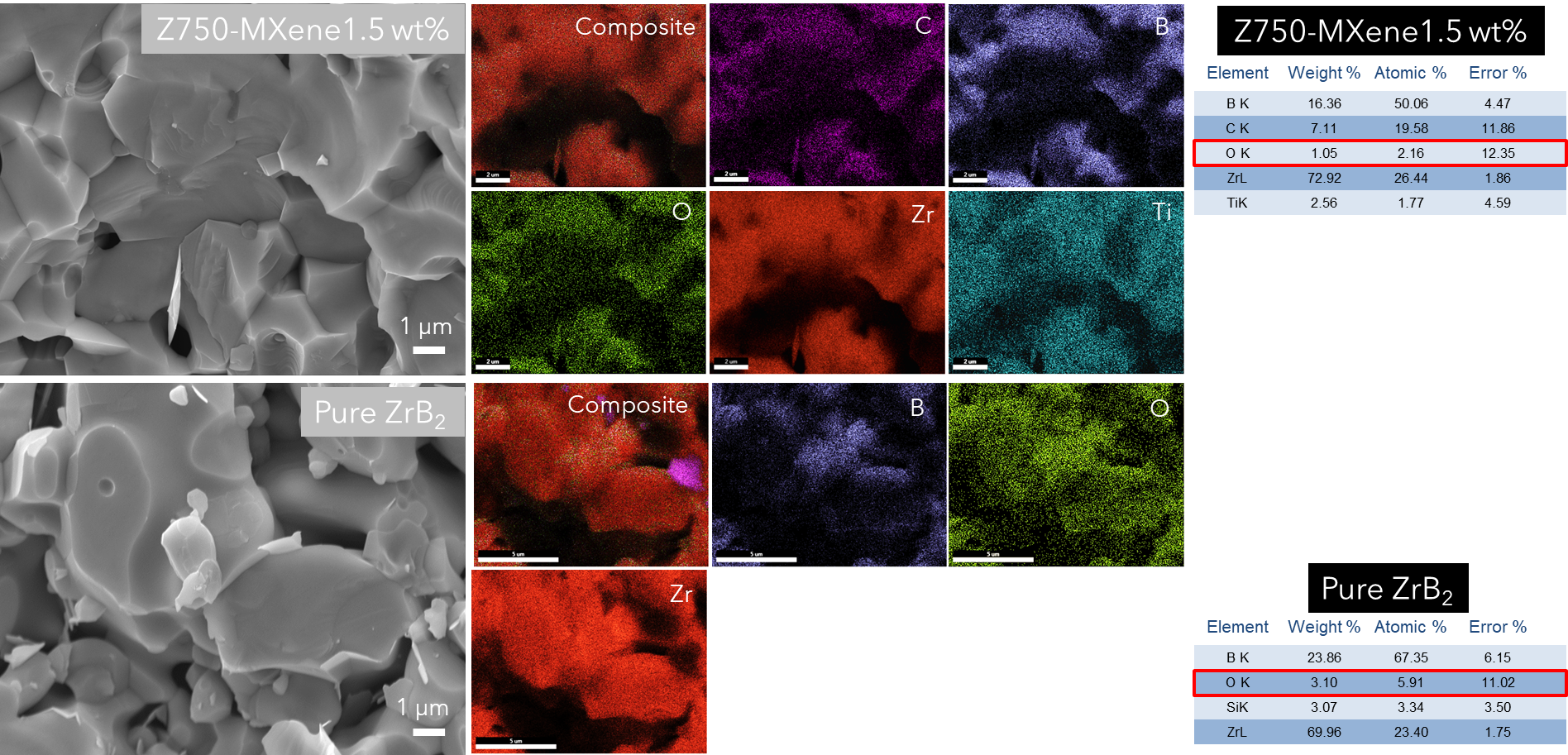
**

**Figure S9:** SEM micrographs and EDS mapping of ZrB_2_-MXene (1.5 wt.%) and pure ZrB_2_ sintered samples. The fractured samples show difference in pore formations with pure ZrB_2_ showing greater porosity. The EDS maps show C and Ti content in the samples with 1.5 wt.% MXene loading. The next oxygen content in pure ZrB_2_ shows ~5.9 at.% Oxygen while the sample with MXene shows the O content at ~2 at.% (Magnification: 3000x).

**Figure S10**


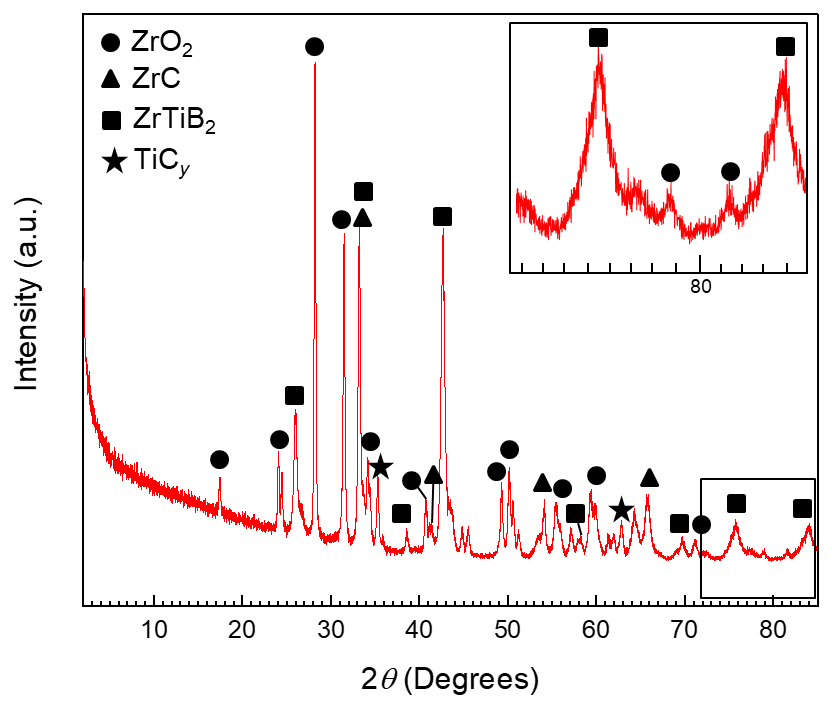


**Figure S10**: XRD pattern of ZrB_2_-MXene (15 wt.%) sintered sample. Note: the sintering cycle is identical to other samples with varying MXene content. (2-step sintering at 1200-1900 ºC).

**Table S1**: Estimated lattice parameters from X-ray diffraction and properties of MXene-UHTCs


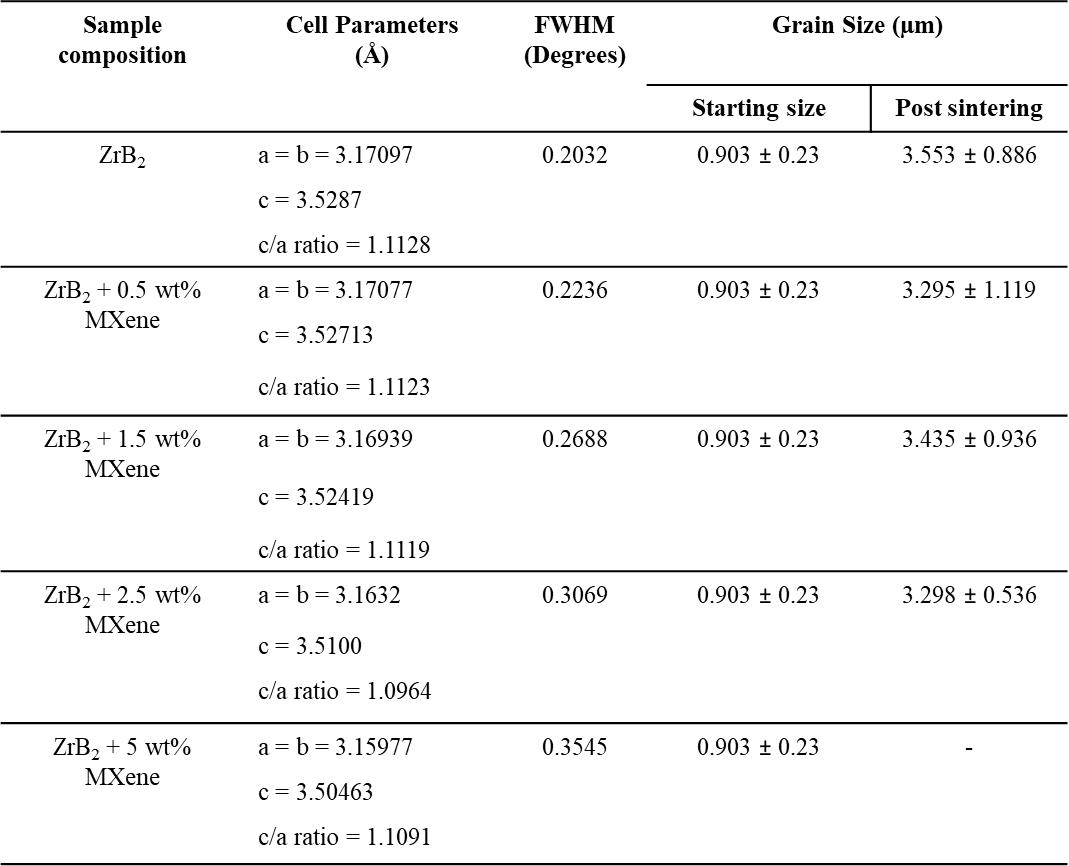


**Figure S11**

**Figure S11**: FWHM vs MXene wt.% fraction in sintered samples.

**Figure S12**


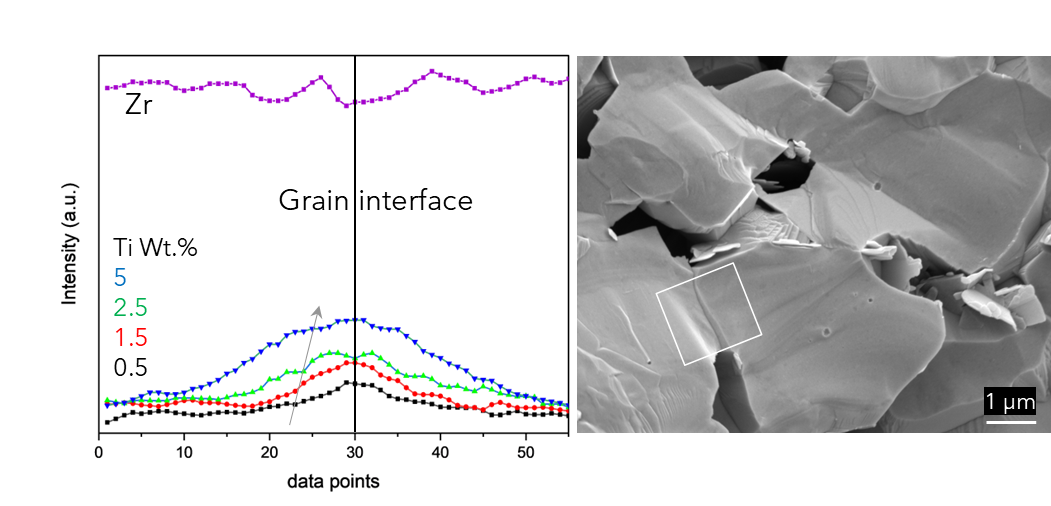


**Figure S12:** EDS point scans across an area of ~ 3 µm^2^ of the composites with the increase in MXene wt.%. The black vertical line indicates the grain boundary interface from where the scans were taken. Scans were performed across straight lines with an average spot size of 2 nm. 50-point scans were performed across a line of length ~ 1 µm. 20 such lines were scanned across the grain boundary interface to normalize the EDS data for each wt.% sample.

**Figure S13**

**
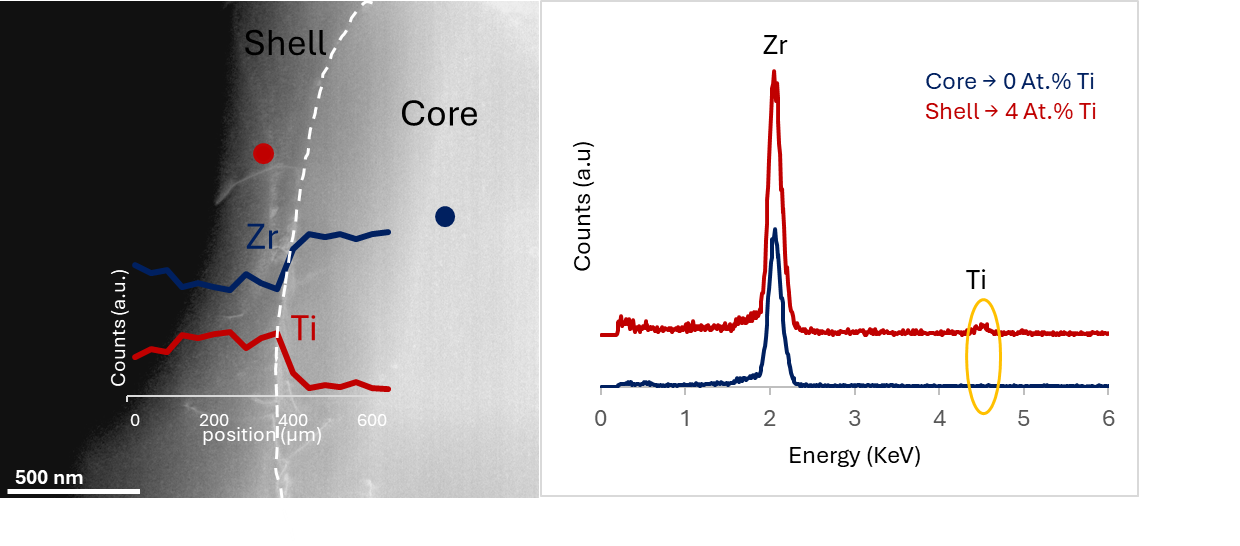
**

**Figure S13:** EDS line scan of the core-shell interface.

**Figure S14**


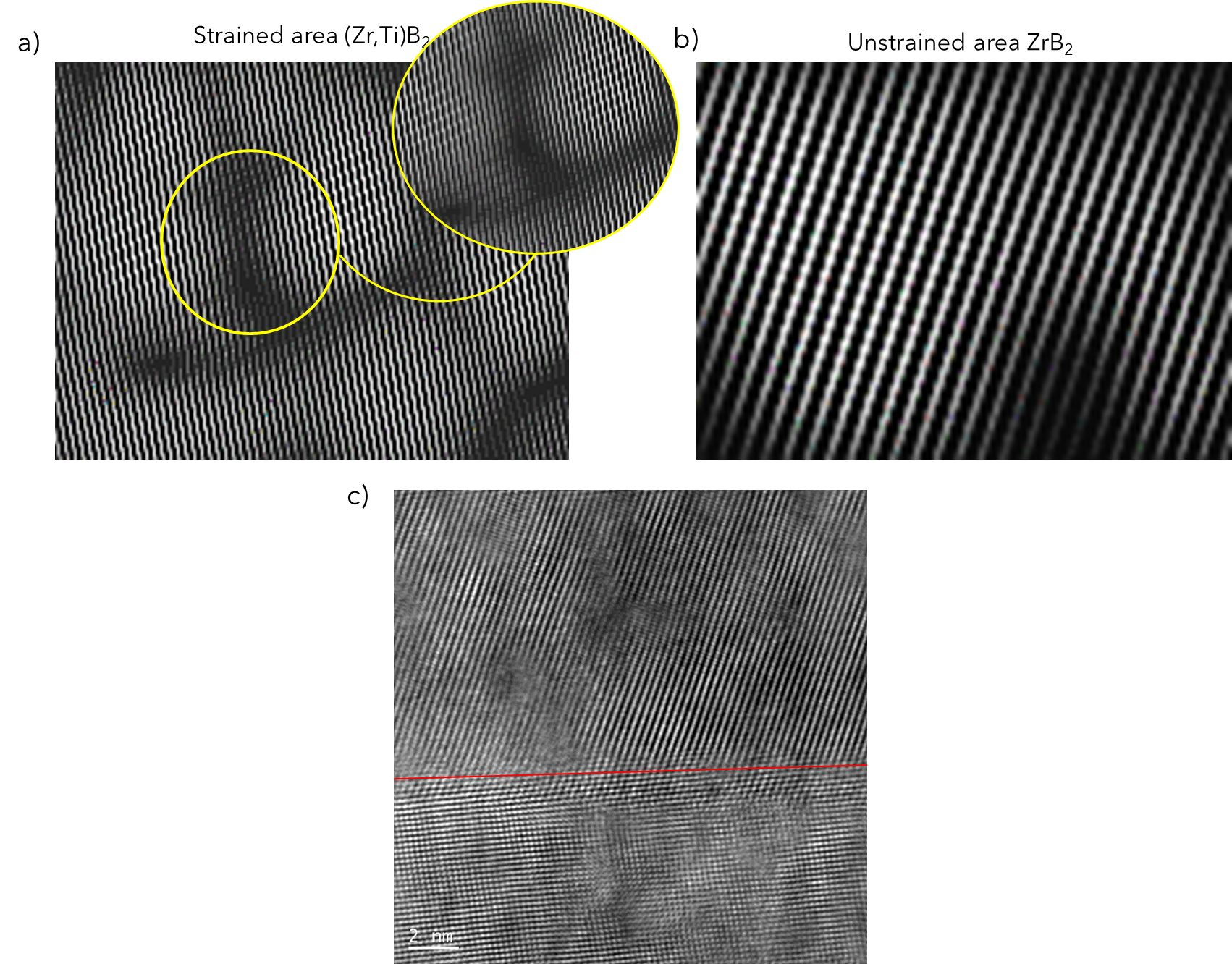


**Figure S14**: a,b)Inverse FFT images of figure 5c showing developed strain in the solid-solution regions of the UHTC, c) TEM micrograph of the interface (bottom). Red line represents the interface boundary.

**Figure S15**

**
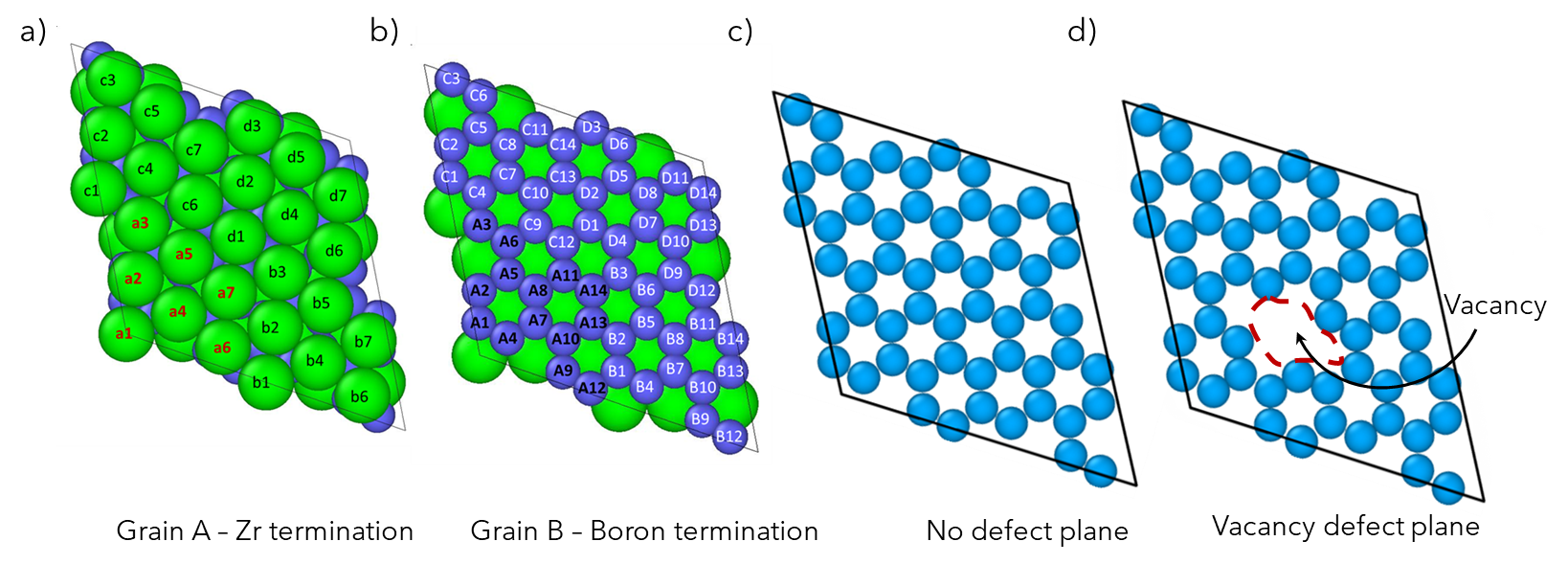
**

**Figure S15**: a,b) The grain boundary unit cell showing Zr (green) and B atom (blue) planes, unit cell showing c) no defect, d) with a vacancy defect.

**Supplementary text S15**

Density functional theory (DFT) calculations were completed in the Vienna ab initio simulation package (VASP)^6^, using the projector augmented wave method (PAW)^7^ and the Perdew, Burke, and Ernzerhof (PBE) exchange correlation functional^8^. The plane wave basis set was selected with a 520-eV cutoff. Using the conjugate gradient method, geometry relaxation was executed with a 0.002 eV/Angstrom force threshold on the ions and a 10e-6 eV threshold energy for electronic self-consistent convergence. Due to the large supercell in real space, the Brillouin Zone was sampled with a single k point centered at gamma. The utilized cell dimensions were 16.78 x 16.78 x 50.52 angstrom. The grain boundary structures were created using the integrated GUI created by Zheng *et al.*^9^ This created a sigma 7 grain boundary (GB) unit cell with a 21.79-degree twist and coherent periodic boundary conditions. **Figure S15 a-b** show the grain boundary sites with labels in the Zr and boron planes.

Due to the periodic boundary conditions each cell contains two grain boundaries, one between the A grain and the B grain and then one between the B grain and the periodic image of the A grain. The grain boundary unit cell was replicated into a 2 x 2 x 7 supercell to reduce the impact of self-interaction of grain boundaries and defects. Smaller and larger replicated supercells were tested, and the 2x2x7 had minimal extraneous interaction while remaining computationally feasible. This supercell was then relaxed to give a baseline of the expected energy levels. From this relaxed configuration, Ti atoms were substituted for Zr atoms in the bulk and at the interface. A single Ti substitution corresponds to 1 out of 28 atoms. Several locations were selected to survey the complete area of possible segregation energies.

The segregation energy (E_segregation_) was calculated using the following equation:

E_segregation_ = E_grain-boundary_ – E_bulk_

Where E_grain-boundary_ is the system energy with a substitution at the grain boundary and E_bulk_ is the system energy with an atomic substitution in the bulk structure.

We performed simulation studies to delineate the diffusion propensity of the Ti atom across the grain boundary (GB) of ZrB_2_. The substitution of a Ti atom at a vacancy site introduced in a ZrB_2_ structure can provide us with a fair understanding of the diffusion pathways leading to the formation of a solid solution “shell” interface in the composite.

The first simulations were to establish a baseline of the system's energy with a single substitution. A Ti substitution replaced a Zr atom in the relaxed grain boundary structure and the cell was allowed to relax further. Several sites were surveyed to assess the range of values. The original coincidence site lattice (CSL) unit cell contained 7 Zr atoms and was replicated twice in the in-plane directions. The labels were selected to reflect this. The A designations are assigned to the rotation center of unit cell. This means that those atoms are in the same position as they would be in the unrotated configuration (directly over the boron ring of the lower grain). These A sites are further identified as a higher symmetry site. The B designation signifies the rest of the unit cell, while C shows the equivalent atom in another unit cell. The segregation energy at each of these sites was determined.

The next step was to establish a more physical boundary by seeding it with vacancies and then estimating the substitutional energy of the Ti atoms at these vacancy sites.

We created a defected grain boundary of ZrB_2_. This involved removing a ZrB_2_ unit cell from the grain boundary to keep it stoichiometric. The atoms were selected close to one another (as shown in **figure S15 a,b**) to ensure distortion in the grain boundary. In each case, the selected atoms were removed, and the structure relaxed again.

From the relaxed structure, a Ti substitution was introduced at a5 and c6 for the d1 case and at d1 and c6 for the a5 case (**figure S15 c, d**). In both cases the stoichiometry is maintained so there is always the same number of Zr, B, and Ti atoms. This means that the Ti is not allowed to fill the vacancy of the Zr but they are adjacent. The segregation energy was calculated for each case and is reported in the following table.

**Table S2**: Segregation energies of Ti substitution at different sites in the defected twist grain boundary

| **Case** | **Substitution** | **E _Segregation_** |
| --- | --- | --- |
| A5-A11-A14 | d1  c6 | -0.2034  -0.2674 |
| D1-A11-A14 | a5  c6 | 0.3465  0.0520 |

**Figure S16**


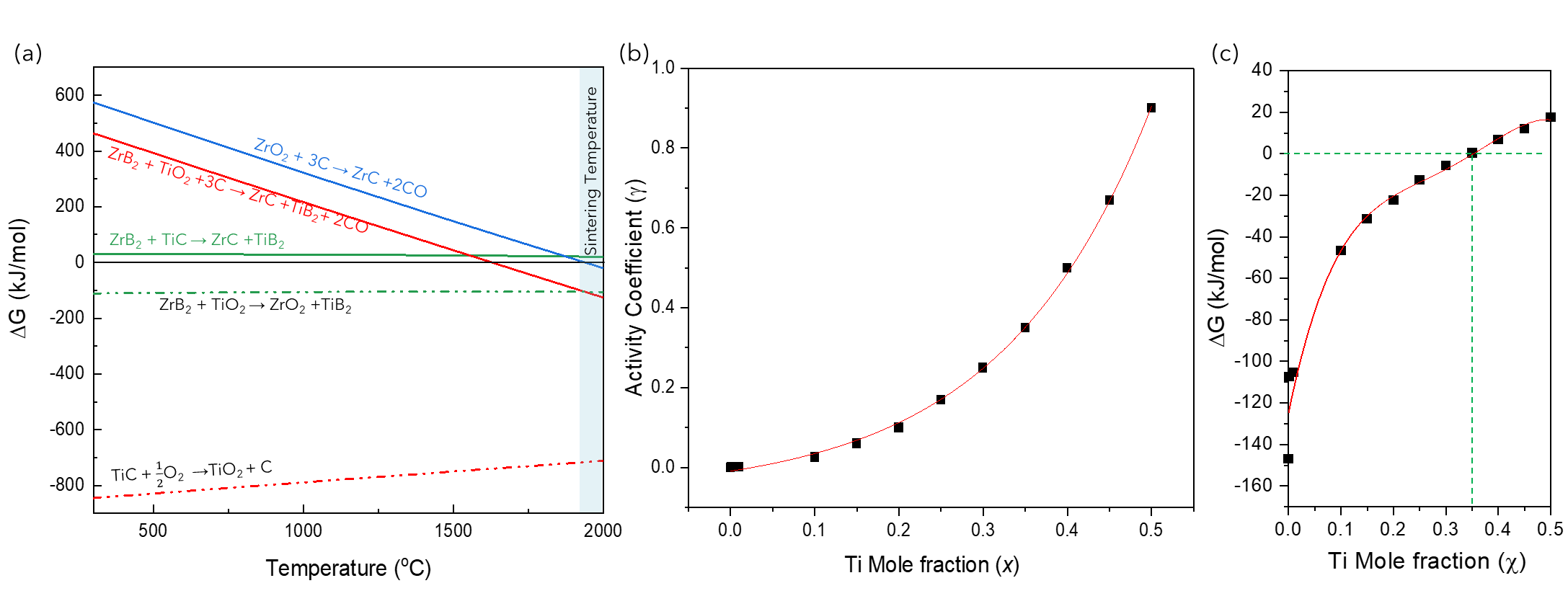
**Figure S16**: a) Gibbs free energy as a function of temperature for various reaction pathways in the ZrB_2_-TiC system, b) activity coefficient vs Ti mole fraction plot, c) Gibbs free energy vs Ti (mole fraction) showing high solubilities established using Debye-Huckel relation.

**Supplementary text S16**

Thermodynamic calculations of the various constituent compounds in the ZrB_2_-TiC systems are shown in **figure S16-a**. The Gibbs free energy values were estimated using THERMOCALC software for all probable reaction pathways of the reactant phases. We assumed that the reactants are ideal stoichiometric compounds and the carbon or boron vacancies in the sublattice were not considered in the energy calculations. The variation in Gibbs free energy (ΔG*_0_*) as a function of temperature indicates that the reaction $ZrB_{2}+TiC\to ZrC+TiB_{2}$ is not feasible in the temperature range 500 to 2000 °C. The estimated Gibbs free energy for the reaction is 21 kJ/mol.

From our XRD analysis, we attributed a shrinkage in lattice of the ZrB_2_ phase due to the formation of a solid solution, where TiB_2_ is present in the form of a mixed crystalline phase along with ZrB­_2_. Previous studies on bulk ZrB_2_ and carbide phases have suggested a partial reaction progression where the energy balance for the formation of mixed solid solutions were estimated via modified partial reactions^[6]^. Similar model may be used to study the reaction pathway of MXene with ZrB_2_ towards formation of a solid solution phase because of titanium diffusion into ZrB_2_: $ZrB_{^{2}}+xTiB_{2}\to Zr_{1-x}Ti_{x}B_{2}$and the Gibbs free energy of the solid solution phase maybe estimated as:

Where ΔG*_ss_* is the Gibbs free energy of the mixed solid solution phase, R is the universal gas constant = 8.314 kJ/mol, T is the temperature, x is the mole fraction of the constituent compound, and $\gamma$ = the activity coefficient of reactants. The activity coefficient of Ti and Zr was assumed to be 1 for pure stoichiometric phases. The variation in the activity coefficient for Ti during diffusion was estimated to be exponential, following Arrhenius principle shown in **figure S16-b**. The variation in the Gibbs free energy for the modified reaction pathway (**figure S16-c**) indicates a higher diffusion limit ~ 35 at.% of Ti into the ZrB_2_ matrix.

The estimated total Gibbs free energy for the solid solution phase is then estimated as a summation of the corresponding energies of formation i.e.: ΔG*_ss_* + ΔG_0_.

We note that the non-stoichiometric nature of MXene which forms TiC*_y_* phase upon transformation, has a greater propensity towards diffusion of titanium in the metallic sublattice of the matrix; while an equally probable diffusion of boron into the non-metallic sublattice is favorable during the initial stages of the reaction when boron oxide is formed as a byproduct. Non-stoichiometric carbide can reduce boron oxide subsequently forming boron carbide phase and carbon mono-oxide as a byproduct, indicating that MXene may be a reducing agent, aiding in eliminating any oxides.

MXenes due to their surface functional moieties including oxygen terminations have not been considered for high temperature applications so far due to their propensity to degrade to titanium oxide when exposed to air. However, we did not observe the formation of titanium oxide in the sintered samples via XRD and TEM analysis. Therefore, we calculated the free energies of the possible reaction pathways of ZrB_2_ with TiO_2_ and ZrO_2_. The formation of TiO_2_ is feasible along the temperature range of 500-2000 °C via the following reaction:

$$TiC+\frac{1}{2}O_{2}\to TiO_{2}+CO\uparrow$$

Further, the propensity of the following reaction between TiO_2_ and ZrB_2_ towards formation of zirconium oxide, $TiO_{2}+ZrB_{2}\to TiB_{2}+ZrO_{2}$ indicates that any available titania further leads towards formation of zirconium oxide in the temperature range (500-2000 °C). The identification of ZrO_2_ in greater fractions at higher MXene weight loadings (5 wt.% and above) supports the hypothesis that zirconia is formed due to reduction of any titanium oxide present in the material.

We also evaluated the effect of excess carbon to evaluate if a reducing environment can prevent formation of ZrO_2_. Estimation of free energies indicates that the presence of a reducing agent, and simultaneous increase in sintering hold times at 1900 °C further prevents formation of any oxides during sintering via the following reaction

$ZrO_{2}+3C\to ZrC+2CO$( ΔG_0_ = -21 kJ/mol @ 1870 °C).

**Figure S17**


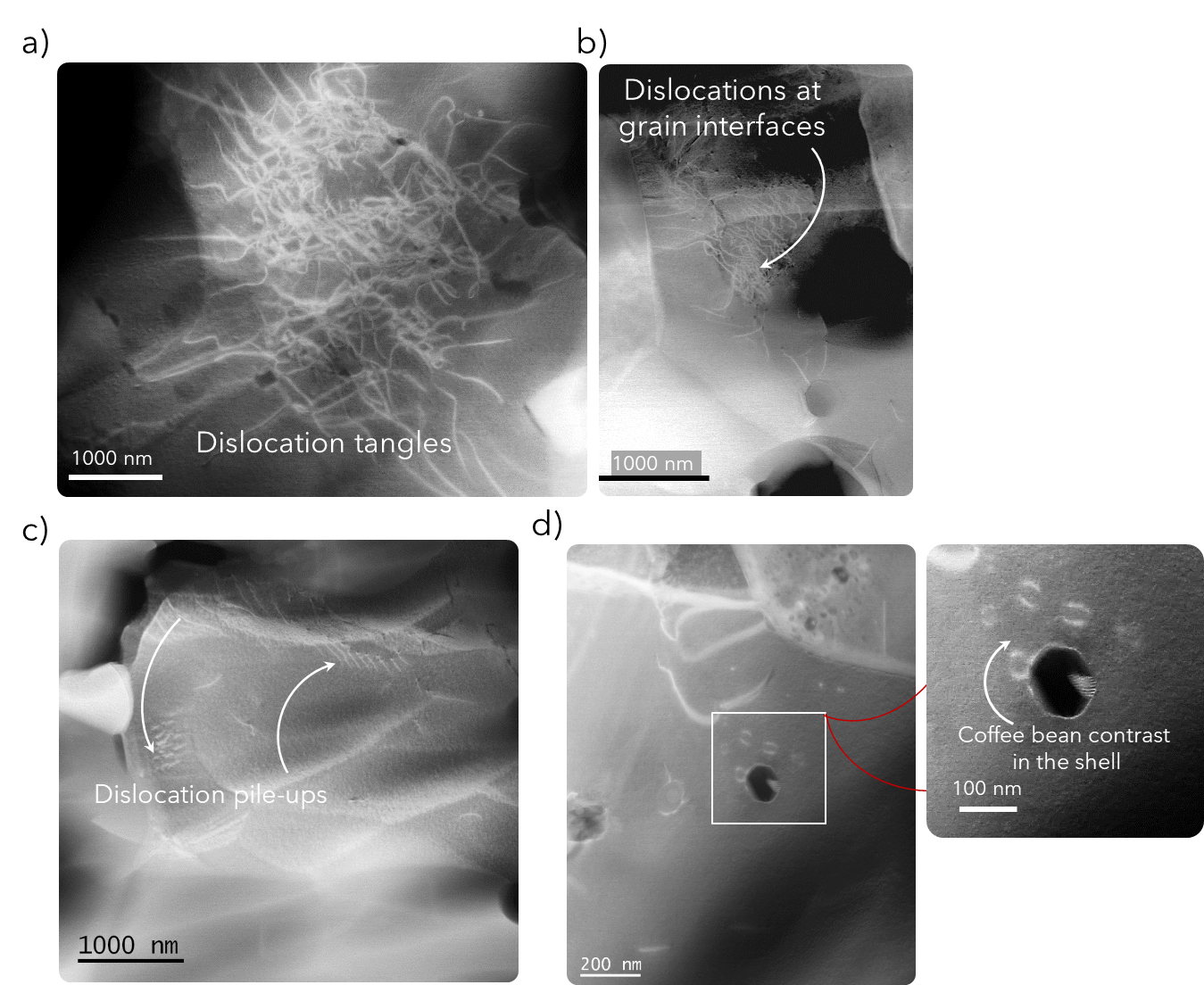


**Figure S17:** TEM micrographs of  ZrB_2_-MXene  (1.5 wt.%) sintered sample  showing a) dislocations tangles and b) along the core-shell, c) dislocations pile-up at the shell interface, d) coffee bean contrast observed in the shell, typically indicating faults rising due to 2D-3D transition during sintering.

**Figure S18**

**
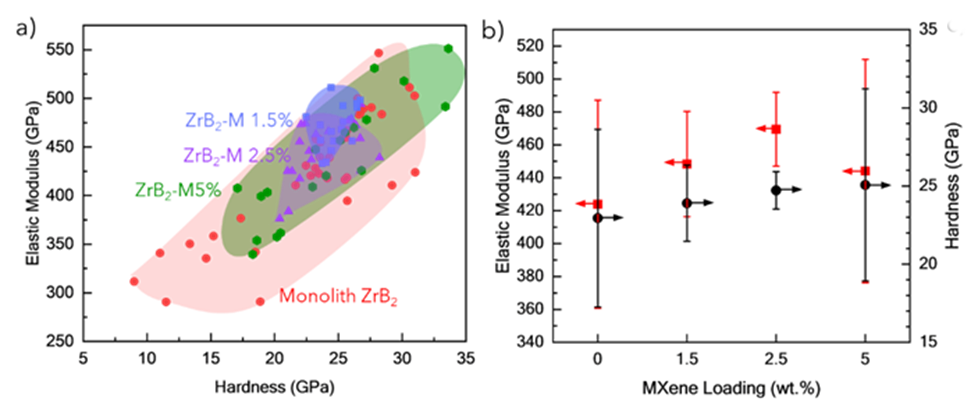
**

**Figure S18:** a) Elastic modulus vs hardness spread for all data points measured on sintered samples characterized via nano-indentation at 10 mN load, showing the spread of values, b) dot plot of hardness, eleastic modulus vs MXene loadings.

**Table S3:** Mechanical properties of MXene-ZrB_2_ UHTCs

| **Composition** | **Hardness**  **GPa** | | **Youngs’s Modulus**  **GPa** | **Fracture Toughness**  **MPa.m^1/2^** |
| --- | --- | --- | --- | --- |
|  | **Nanoindentation** | **Vickers** |  |  |
| ZrB_2_ | 22.94 ± 5.68 | 19.42 ± 4.41 | 423.90 ± 63.22 | 2.08 ± 0.50 |
| ZrB_2_-Ti_3_C_2_ (1.5 wt.%) | 23.89 ± 2.43 | 23.01 ± 1.20 | 448.34 ± 31.97 | 2.63 ± 0.27 |
| ZrB_2_-Ti_3_C_2_ (2.5 wt.%) | 24.71 ± 1.19 | 25.10 ± 1.57 | 469.46 ± 22.04 | 2.97 ± 0.44 |
| ZrB_2_-Ti_3_C_2_ (5 wt.%) | 25.06 ± 6.14 | 22.31 ± 3.42 | 444.06 ± 67.88 | 2.51 ± 0.28 |

**Figure S19**

**
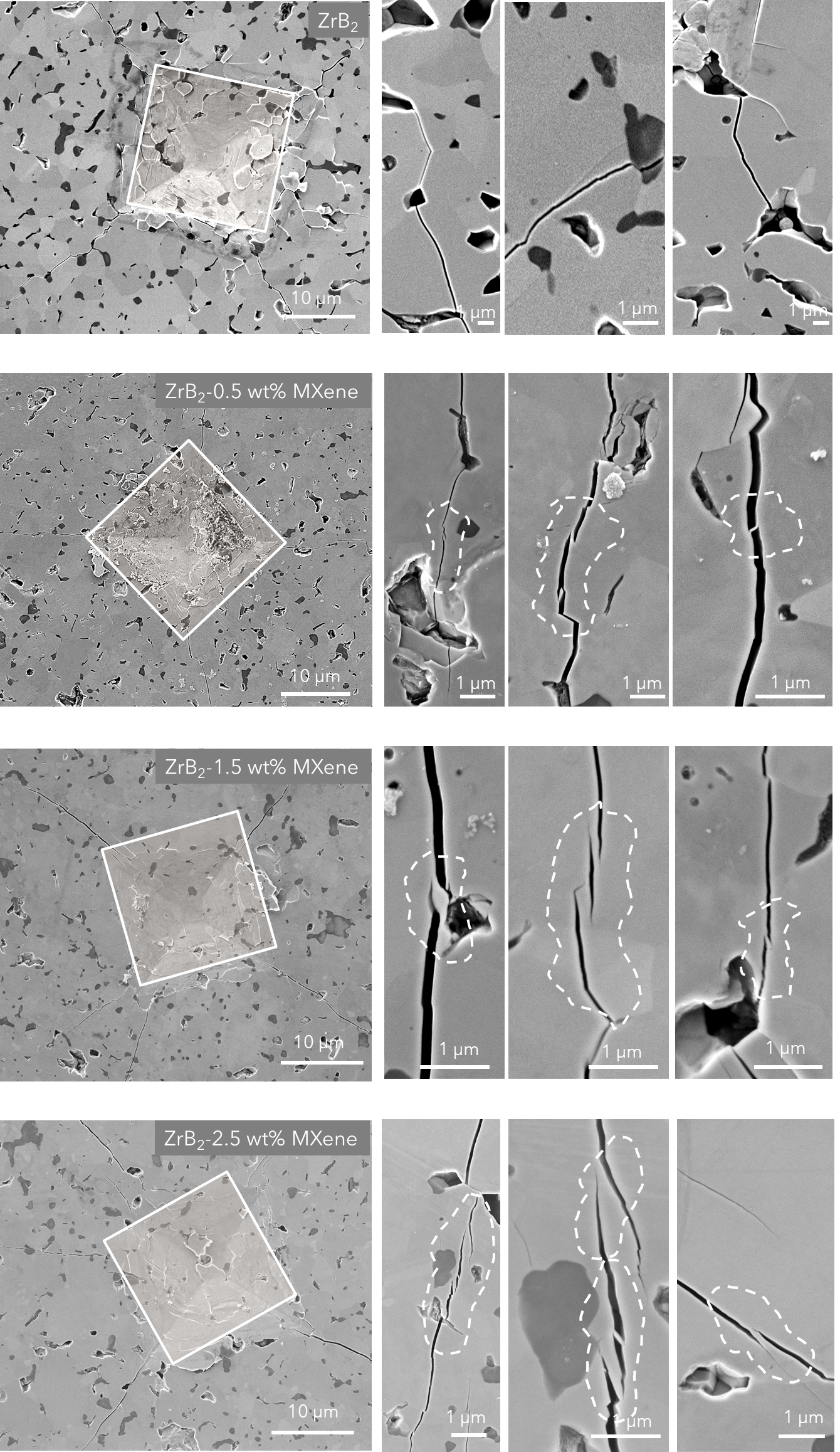
**

**Figure S19:** Indentation and crack propagations in ZrB_2_  and ZrB_2_ MXene UHTCs.

**Table S4**: Flexural strength of sintered samples

| **Composition** |  |  | Flexural Strength (MPa) |  |  |
| --- | --- | --- | --- | --- | --- |
|  | **S1** | **S2** | **S3** | **S4** | **S5** |
| ZrB_2_ | 233.67 | 279.95 | 245.88 | 234.4 | 241.6 |
| ZrB_2_+Ti_3_C_2_ (1.5 wt.%) | 319.31 | 315.15 | 399.7 | 341.1 | 387.2 |
| ZrB_2_+Ti_3_C_2_ (2.5 wt.%) | 352.44 | 337 | 401.12 | 379.91 | 400.03 |

**Figure S20**


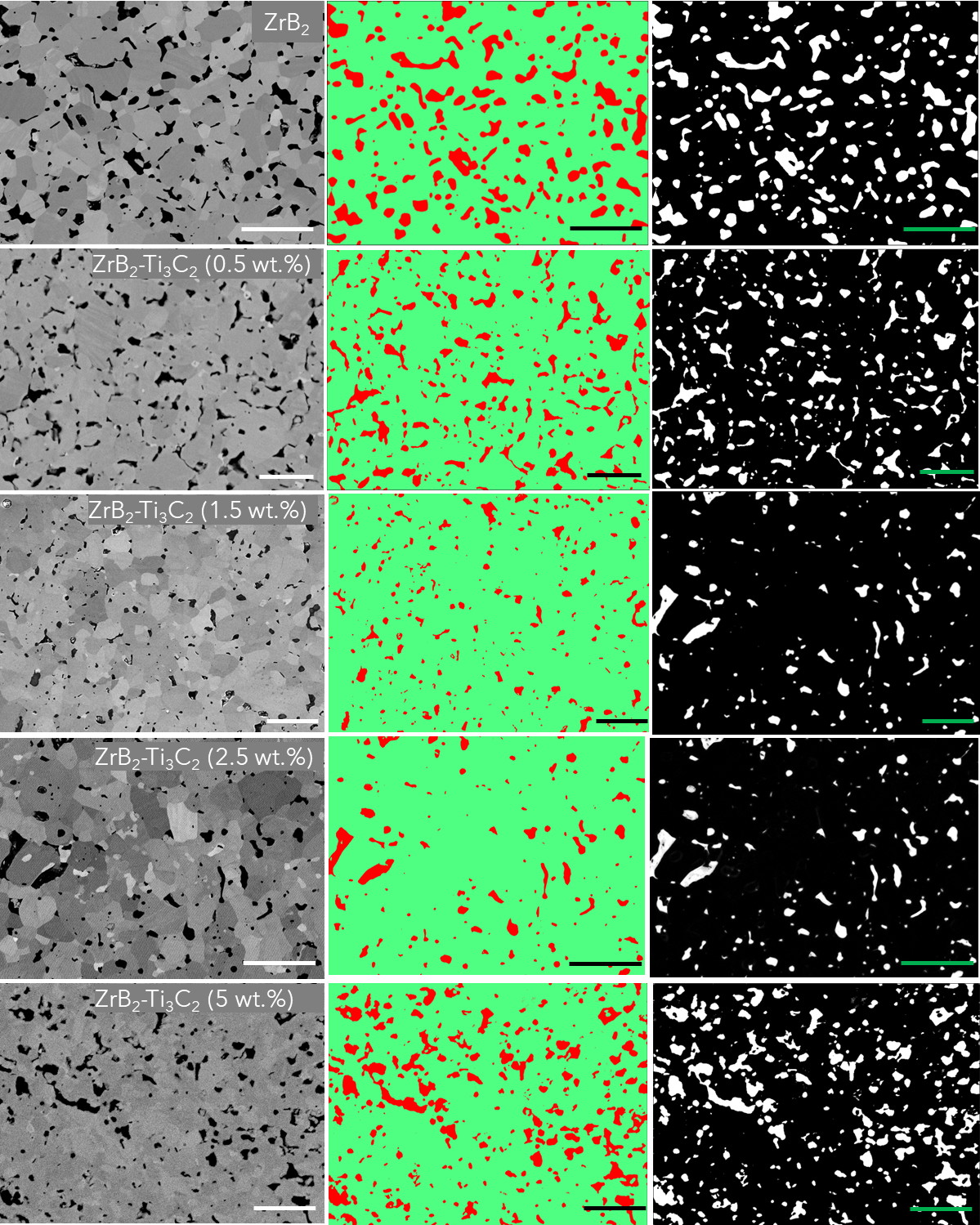


**Figure S20:** Image segmentation showing porosity of the sintered ZrB_2_-MXene samples. scale bar = 10 microns. Area in green (and black in monochrome) represents sintered zones, area in red (and white) indicate pores.

**Table S5**: Image segmentation data for estimation of porosities in sintered samples.

| **Composition** | **Pixel Area** | **Dark region** | **Bright Region** | **Null Area (%)** |
| --- | --- | --- | --- | --- |
| ZrB_2_ | 1221120 | 174657 | 1046463 | 14.30302 |
| ZrB_2_+Ti_3_C_2_ (0.5 wt.%) | 473620 | 55609 | 418011 | 11.74127 |
| ZrB_2_+Ti_3_C_2_ (1.5 wt.%) | 1214760 | 73324 | 1141436 | 6.036089 |
| ZrB_2_+Ti_3_C_2_ (2.5 wt.%) | 4953952 | 348145 | 4605807 | 7.027622 |
| ZrB_2_+Ti_3_C_2_ (5 wt.%) | 4865400 | 685824 | 4179576 | 14.09594 |

**References**

[1] B. C. Wyatt, S. K. Nemani, K. Desai, H. Kaur, B. Zhang, B. Anasori, *Journal of Physics: Condensed Matter* **2021**, 33, 224002.

[2] J. M. Lonergan, W. G. Fahrenholtz, G. E. Hilmas, *Journal of the American Ceramic Society* **2015**, 98, 2344.

[3] D. Kalish, E. V. Clougherty, *Journal of the American Ceramic Society* **1969**, 52, 26.

[4] M. A. Hope, A. C. Forse, K. J. Griffith, M. R. Lukatskaya, M. Ghidiu, Y. Gogotsi, C. P. Grey, *Phys Chem Chem Phys* **2016**, 18, 5099.

[5] P. P. Michałowski, M. Anayee, T. S. Mathis, S. Kozdra, A. Wójcik, K. Hantanasirisakul, I. Jóźwik, A. Piątkowska, M. Możdżonek, A. Malinowska, R. Diduszko, E. Wierzbicka, Y. Gogotsi, *Nature Nanotechnology* **2022**, 17, 1192.

[6] V. I. Ivashchenko, P. E. A. Turchi, V. I. Shevchenko, N. R. Mediukh, L. Gorb, J. Leszczynski, *Materials Chemistry and Physics* **2021**, 263, 124340.
